# Temperature Dependence of Charge and Spin Transfer in Azurin

**DOI:** 10.1021/acs.jpcc.1c01218

**Published:** 2021-04-29

**Authors:** Yutao Sang, Suryakant Mishra, Francesco Tassinari, Senthil Kumar Karuppannan, Raanan Carmieli, Ruijie D. Teo, Agostino Migliore, David N. Beratan, Harry B. Gray, Israel Pecht, Jonas Fransson, David H. Waldeck, Ron Naaman

**Affiliations:** † Department of Chemical and Biological Physics, Weizmann Institute, Rehovot 76100, Israel; ‡ Department of Chemical Research Support, Weizmann Institute of Science, Rehovot 76100, Israel; § Department of Chemistry, Duke University, Durham, North Carolina 27708, United States; ∥ Department of Chemical Sciences, University of Padova, Via Marzolo 1, Padova 35122, Italy; ⊥ Beckman Institute, California Institute of Technology, Pasadena, California 91125, United States; # Department of Immunology, Weizmann Institute, Rehovot 76100, Israel; ∇ Department of Physics and Astronomy, Uppsala University, Uppsala 752 36, Sweden; ◆ Department of Chemistry, University of Pittsburgh, Pittsburgh, Pennsylvania 15260, United States

## Abstract

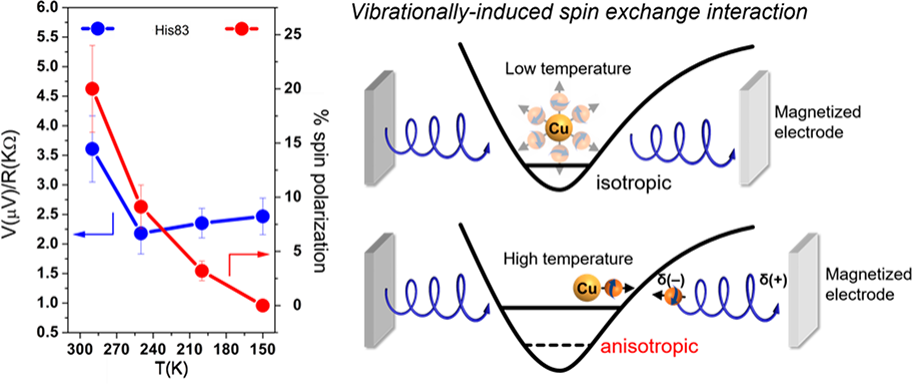

The steady-state charge and spin transfer yields were measured for three different Ru-modified azurin derivatives in protein films on silver electrodes. While the charge-transfer yields exhibit weak temperature dependences, consistent with operation of a near activation-less mechanism, the spin selectivity of the electron transfer improves as temperature increases. This enhancement of spin selectivity with temperature is explained by a vibrationally induced spin exchange interaction between the Cu(II) and its chiral ligands. These results indicate that distinct mechanisms control charge and spin transfer within proteins. As with electron charge transfer, proteins deliver polarized electron spins with a yield that depends on the protein’s structure. This finding suggests a new role for protein structure in biochemical redox processes.

## Introduction

Theoretical and experimental studies have taught us how structure, energetics, and dynamics impact biological electron transfer kinetics. For example, studies of metal-labeled proteins^1,2^ revealed how their polypeptide matrices direct charge transfer over large distances by thermally activated tunneling.^3−13^ Limited attention has however been paid to the spin of the electron as it transits within a protein.

Recent studies documented spin-selective electron transfer and transport through chiral (bio)molecules.^14,15^ Electron transmission through chiral molecules is found to depend on the electron’s spin orientation (the chiral-induced spin selectivity or CISS effect).^16^ Spin-selective electron transport has been measured via peptides and proteins,^17^ in multiheme cytochromes,^18^ and via membranes containing bacteriorhodopsin.^19^ Here, we show that spin-selective intramolecular electron transfer via the blue copper protein azurin labeled by Ru(II)(bpy)_2_(im) (bpy = 2,2′-bipyridine, im = imidazole) on specific surface histidine residues is thermally activated. The employed azurins were modified in a way that its electron transfer is activationless, which enabled to distinguish between the temperature dependence of spin filtering and that of the charge transfer.

Ru-modified proteins have proven most valuable for investigating protein-mediated electron transfer mechanisms,^3,4,20−23^ yet earlier studies have not addressed the spin filtering that may accompany electron transfer. The methods described here allow the study of both charge and spin transfer via the Ru-modified azurins. Native and mutated surface histidine residues of azurin have been derivatized with Ru-bipyridyl complexes, enabling the initiation of photoinduced electron transfer between the surface Ru complex and the protein’s Cu(II) site. The known surface histidine positions provided variation of the copper to ruthenium separation distance, coupling pathways, and charge-transfer kinetics. Signatures of the photoinduced electron transfer and spin polarization were measured as a function of temperature for three azurin derivatives (His83, 124, and 107)^6^ adsorbed on a silver substrate, which forms the top contact in a magnetic tunnel junction (MTJ) device and allows measurement of both charge and spin transfer.^24^

**Figure 1:**
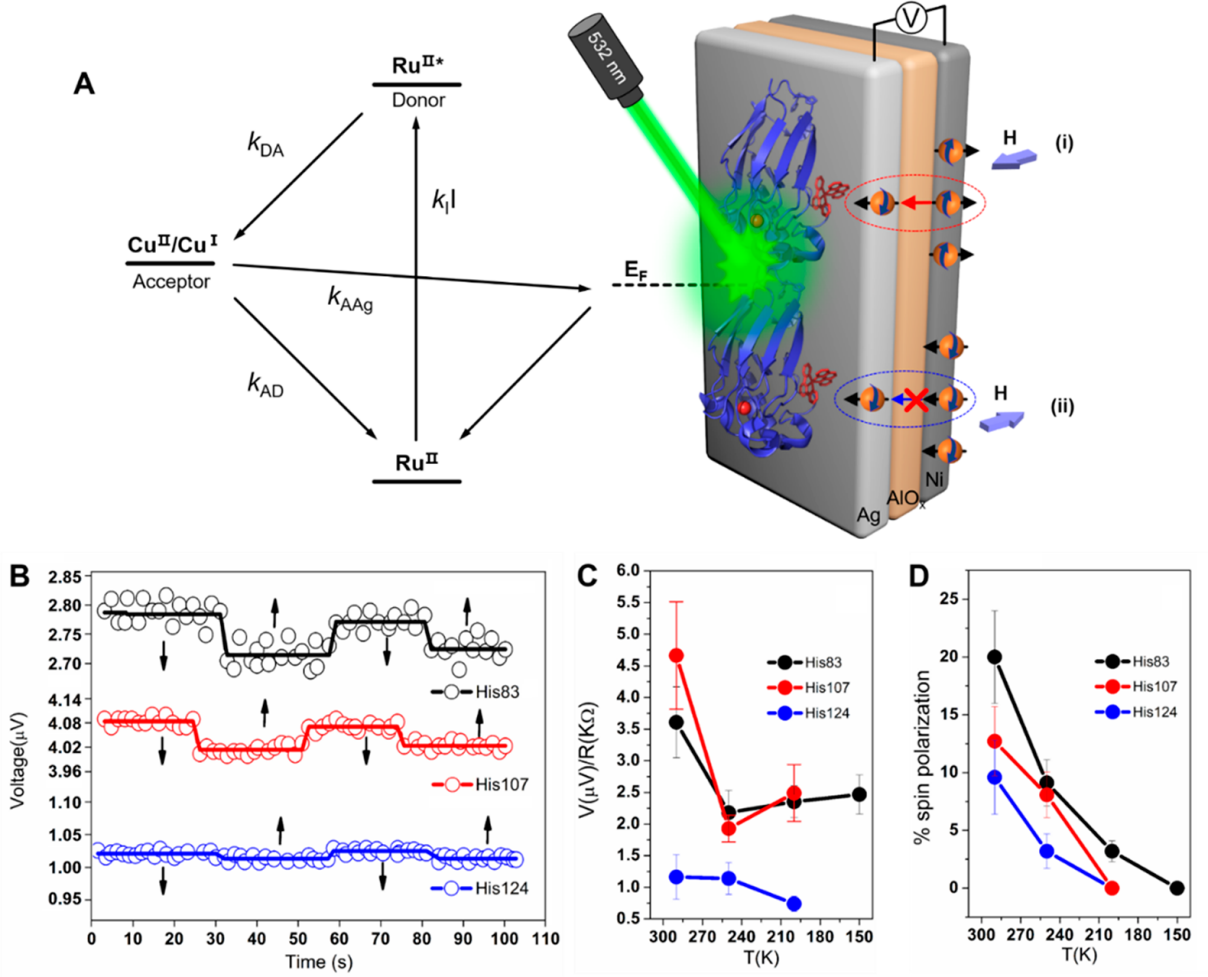
Measurements with MTJ device. (A) Scheme for measuring the temperature-dependent electron and spin transfer from dry films of Ru-modified azurin to a silver layer. Energy-level scheme of the device for measuring photoinduced charge transfer and the adsorbed molecules containing donor (Ru(II)*) and acceptor (Cu(II)) groups, adjacent to the silver film (gray). A laser of intensity *I* is used to excite the donor. *E*_F_ is the Fermi level of the device without a magnetic field applied to the nickel. Upon photoexcitation, an electron is excited on the Ru ion at a rate of *k*_I_I, and an electron from the silver is transferred to the hole left on the Ru. The excited electron is transferred to the Cu (II) ion (*k*_DA_) and from there back to the silver substrate (*k*_AAg_). The arrows indicate the electron transfer processes giving rise to a photovoltage. (B) Time profiles of the photovoltage between the silver and nickel layers exposed to magnetic fields pointing toward or away from the protein layer (black arrows); measurements are at 290 K with monolayers of His83 (black), His124 (blue), and His107 (red). (C) Temperature dependence of the average (spin-independent) photovoltage as a function of temperature. (D) Percentage spin polarization as a function of temperature.

An illustration of the MTJ device is shown in Figure 1A. Photoexcitation of the Ru-bipyridyl species in the modified azurin film energizes intramolecular electron transfer (between Ru and Cu) and delivers charge to the Ag electrode in a second step. Under steady-state illumination, the photovoltage produced on the Ag electrode reports on the quantum yield of charge transfer, while the steady-state excess charge produced as a function of the MTJ magnetization allows measurement of the electron spin population on the Ag (the spin polarization). The experimental results show distinctly different temperature dependences of the photoinduced charge-transfer yield and the spin polarization (Figure 1C,D). Corresponding studies on electron transport through native azurin in molecular junction devices were performed and show that the spin polarization of the electron current increases as the temperature increases. Thus, while the charge transfer depends only weakly on temperature, spin filtering is thermally activated. In addition to the study of the photoinitiated electron transfer within the protein, two complementary measurements were performed. In the first, the spin-dependent polarizability of the protein was confirmed by Hall device measurements (see the Supporting Information and refs (18) and (25)). In the second, spin-dependent conduction was measured through a film of azurin in a spin valve configuration as a function of temperature.^26−28^

It is proposed that the temperature-activated spin filtering arises from anharmonic vibrations at the copper ion coordination site, which induce a spin-dependent exchange interaction that increases with increasing temperature. Previous EPR studies are consistent with this proposed hypothesis. The long spin polarization lifetime (typically microseconds) suggests that biomolecules could use spin filtering to direct redox processes that are driven by electron transfer.

## Methods

### Protein

*Pseudomonas aeruginosa* azurin (native His83) and two mutants where histidine have been introduced by single-site mutations at positions 107 and 124 were labeled by Ru(II)(bpy)_2_(im) (bpy = 2,2′-bipyridine, im = imidazole). Preparation and purification of all three labeled azurins were as described previously.^6,23^ Successful labeling was confirmed by UV–vis spectroscopy and mass spectrometry.

### Preparation of MTJ Device

Using the MTJ device described in Figure 1A, we studied electron and spin transfer via three different azurin Ru-derivatives as a function of temperature, from 290 to 150 K, and as a function of the magnetic field direction, by applying fields of +0.4 and −0.4 T. All proteins were bound to the silver film through the thiol of a disulfide bond (1,6-hexanedithiol) connecting Cys 3 and Cys 26 to the Ag.

Protein binding to the surface was achieved by exposing the device surface to a phosphate (0.4 M, pH 7.2) buffer solution containing azurin (100 μM) for 2 h; adsorption was verified by XPS (Figure S1) and UV–vis absorption spectroscopy (Figure S2). Following binding, the sample was washed twice with 0.4 M phosphate buffer and then washed with water for 5 s. Having the protein bound to the Ag via Cys3/Cys 26 made the donor (Ru) closer to the surface than the acceptor (Cu). The samples were illuminated by a 532 nm laser (with an intensity of 0.4 mW/cm^2^), and a positive photovoltage was measured indicating that a net transfer of electrons occurs from the electrode to the protein, i.e., from the Ag film to the photogenerated Ru^III^ site. No (steady-state charging) signal was detected for the native azurin, confirming the role of the Ru ion in the photosignal.

### Magnetoresistance Measurements

**Figure 2:**
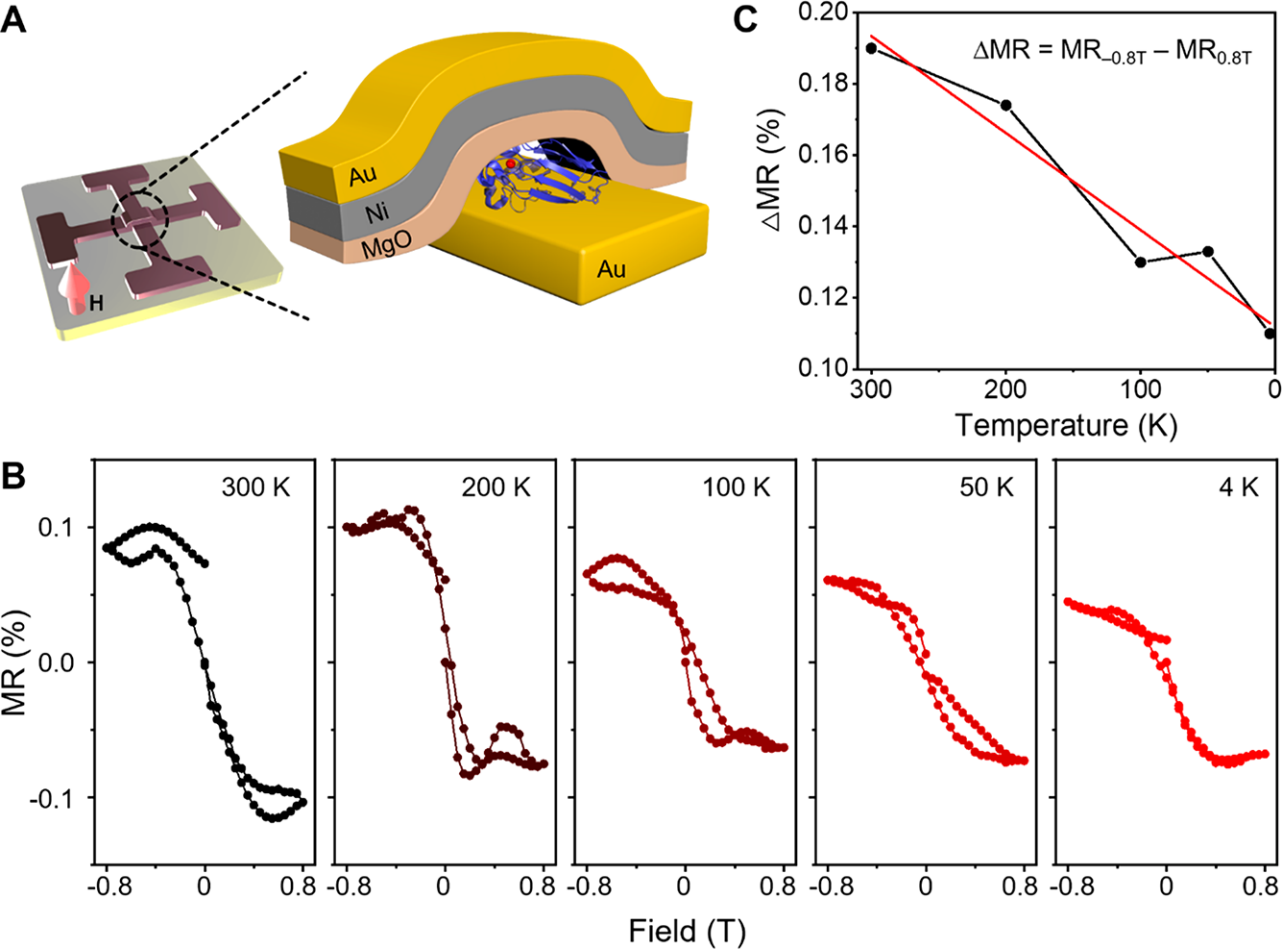
Magnetoresistance measurements of wild-type, native azurin. (A) Schematic illustration of the four-contact device that was used for measuring the temperature-dependent magnetoresistance signal from wild-type, native azurin which is located between gold and ferromagnetic electrodes. (B) Magnetoresistance (MR), reported as a percentage, for a number of discrete fields in the range of −0.8 to 0.8 T and at five different temperatures. (C) Plot of the difference in the magnetoresistance taken at the two extreme fields, ΔMR = MR_–0.8T_ – MR_0.8T_, as a function of temperature.

For the charge and spin transport measurements we apply a four-contact configuration shown in Figure 2, as already described in ref (26). Monolayer preparation followed the same procedure as for the Hall devices (see the Supporting Information, and Experimental Details). All the electrical measurement was carried out within the SQUID-MPMS3 made by Quantum Design. A magnetic field of 0.8 T was applied out-of-plane of the device, and the resistance was measured by a standard 4-probe method. A constant current of 1 mA was applied, and the voltage was measured across the junction using a Keithley SMU model 2400 as a current source and a Keithley nanovoltmeter model 2182A for the voltage measurement. The magneto-resistance (MR) values produced as a function of temperature were calculated by MR(*T*) = , where *R*_M_(*T*) and *R*_0_(*T*) are the resistances measured at magnetic field M and zero, respectively.

### Theoretical Simulations of Electronic Coupling in His83 Ru-Modified Azurin

The electronic structure of the Cu(II) active site was obtained from density functional theory (DFT) calculations at the PBE0/6-31g* level of computational accuracy, using the NWChem program.^29^ This was followed by molecular dynamics (MD) simulations using the NAMD software package^30^ with the AMBER ff14SB force field,^31^ except for the Cu and Ru redox sites, for which the force field parameters were taken from refs (32) and (33), respectively. The TIP3P model^34^ was used to describe the water (further details on the MD simulations are provided in the Supporting Information). The simplifying choice of carrying out MD simulations of the solvated protein is supported by previous studies showing very similar donor-to-acceptor electron transfer rates for azurin molecules in crystals and in solution.^4,7^ The dynamics of the system was simulated for the Ru(II)/Cu(II) charge state, and the MD snapshots were used to study the electron tunneling pathways from the photoexcited Ru center to the Cu center through the Pathway 1.2 plugin.^35^ Since the local electronic excitation at the Ru center is much faster than the nuclear motion and starts from a nuclear conformation corresponding to the ground electronic state of Ru(II), the MD simulations were performed using force field parameters that are appropriate to describe the nuclear dynamics while Ru(II) is in its ground electronic state.

## Results and Discussion

### Experimental Results

#### Photoinduced Charge and Spin Transfer

Figure 1A shows the structure of the MTJ device that measures the photoinduced charge-transfer yields and spin polarization as a function of temperature. The detailed structure of the device is presented in the Supporting Information and in ref (24). The experiment measures the photovoltage generated on the device’s top electrode (Ag) as the magnetization of the lower Ni electrode is varied. Because charge tunneling from the Ag to the magnetized Ni layer is spin-dependent, the photovoltage difference that is generated for the two different Ni magnetization directions (parallel and antiparallel to the surface normal), reports on the Ag electrode’s spin population, which can be ascribed to the spin-selective electron transfer from Az (*vide infra*). The sum of the parallel and antiparallel photovoltage is proportional to the total unpolarized charge-transfer yield. An external magnetic field (0.4 T) is applied normal to the Ag surface, and the voltage difference between the two metal layers is measured under continuous 532 nm laser illumination.

We first consider the case without the magnetic field. Photoexcitation of the Ru chromophore at 532 nm produces a Ru(III)/Cu(I) charge-separated state. An electron from the Ag surface can then be transferred to the hole on the oxidized donor Ru(III) species. Charge recombination from Cu(I) to the metal substrate regenerates the initial state. Under irradiation, steady-state populations of Ru(III) and Cu(I) are created and lead to a photoinduced charging of the Ag electrode. The photovoltage generated between the silver and nickel films is sensitive to the rates of Ru(III)/Cu(I) production, Ru(III) reduction by the Ag, and Cu(II) reduction by electron transfer from the Ag film. Parasitic relaxation processes (such as energy transfer from the Ru(II)excited state to the Ag layer) will reduce the overall yield of charge transferred to the silver substrate. Because of uncertainties in these rates and in the protein surface coverage, our analysis focuses on the temperature dependence of the photovoltage (i.e., the change of the photovoltage with temperature rather than its absolute magnitude).

In the presence of an applied magnetic field, the photovoltage that is measured across the tunnel junction depends on the relative spin population between the electrons of the Ag electrode and those of the Ni electrode. Consider, for example, the case where the Ni is magnetized so that its electron spins are aligned antiparallel with the excess electron spins on the silver electrode (Figure 1A(i)). In this case, the electrons can tunnel more readily through the aluminum oxide, and the voltage that is measured will be lower than that in the case where the Ni is magnetized in the opposite direction (Figure 1A(ii)). Thus, a photovoltage change with the direction of the applied magnetic field indicates a difference in the spin population of electrons on the Ag layer. The data in Figure 1B show that the photovoltage indeed changes with the magnetic field direction. Thus, the photogenerated charge carriers on the Ag layer must be spin-polarized. If the charge-transfer rate constant *k*_DA_ is spin-dependent (Figure 1A), then the hole population created on the Ru sites is spin-polarized, and the electron population transferred from the silver electrode to the hole sites should also be spin-polarized. If the electron transfer from Cu(I) to Ag is spin-polarized, then it can also influence the relative spin populations. The experiment does not distinguish between these two possibilities. The amount of total charge transferred as a function of temperature is indicated in Figure 1C, while the difference in the spin populations generated under the two magnetic fields is reported as a spin polarization in Figure 1D.

Figure 1B shows a time profile of the photoinduced spin-dependent charge transport signals measured on the three different Ru-azurin derivatives at room temperature. The photovoltage magnitude changes upon reversing the direction of the magnetic field from parallel (*V*_↑_) to antiparallel (*V*_↓_) with respect to the surface normal (see schematic in panel A). These data provide the relative charge-transfer yield, *V*_↑↓_ = (*V*_↑_ + *V*_↓_)/2, and the resultant spin polarization, *P* = (*V*_↑_ − *V*_↓_)/*V*_↑↓_. It is difficult to quantitatively compare the charge-transfer yield between the three different protein assemblies because the surface coverage of the bound proteins varies up to 20%, (as judged by XPS (Figure S1) and UV–vis absorption spectroscopy (Figure S2)). Nevertheless, the ratios of photovoltage magnitudes observed for the different derivatives at 290 K are significant, 4.7:3.7:1.2 (His107/His83/His124), as shown in Table 1. This trend in charge-transfer efficiency is different from that of the experimentally measured intramolecular electron transfer rates, which is *k*_DA_(His83) > *k*_DA_(His124) > *k*_DA_(His107) (Table 1).^3^ This difference likely arises from competition between the intramolecular electron transfer rate *k*_DA_ and the quenching of the Ru(II) excited state by energy and electron transfer to the Ag electrode.^36^

**Table 1:** Observed Spin Polarization, Photovoltage, and Intramolecular Electron Transfer Rate Constants, in Relation to the Ru-to-Cu Distance of the Three Mutants

property	His83	His107	His124
Ru-to-Cu distances (nm)	1.7	2.58	2.05
His-to-Cys3/Cys26 distance (nm)	2.03	2.07	1.81
Cu-to-Cys3/Cys26 distance (nm)	2.6	2.6	2.6
spin polarization, *P* (%)	20	12	9
photovoltage, *V*_↑↓_ (μV)	3.7	4.7	1.2
intramolecular rate, *k*_DA_ (s^–1^)	1 × 10^6^	2.4 × 10^2^	2.2 × 10^4^

All the azurins are bound to the Ag by thiol linkage(s) of their Cys3 or Cys26 residues. Hence, the Cu is remote from the Ag electrode, and the His124 derivative has the Ru significantly closer to the Ag surface than do the His83 or His107 derivatives. Control experiments on wild-type azurin (without Ru modification) show a very weak residual photovoltage (∼20 nV) and no magnetic field dependence (Figure S3).

The temperature dependences of the charge-transfer yield and the spin polarization observed for the three azurin derivatives are shown in Figure 1C,D. The average photovoltage was divided by the resistance of the AlO_*x*_ layer which was determined at each temperature. The resulting quantity has the dimensions of a current and is assumed to be proportional to the steady-state charge-transfer yield. The data indicate that the yields change by less than a factor of 2 over a 150° temperature range, and this is consistent with earlier studies that found the photoinduced electron-transfer kinetics to be nearly activationless.^3^Figure 1D shows the spin polarization *P* versus temperature. The spin polarization follows a different trend than the charge-transfer yields, namely *P*(His83) > *P*(His107) > *P*(His124), at 290 K. *P* decreases to zero as the temperature is lowered. The temperature-dependent spin polarization was corrected for phonon quenching (see the Supporting Information for details), based on earlier studies performed with the same device structure for adsorbed DNA.^24^

The temperature dependences of the charge-transfer yield and spin polarization are significantly different. The charge-transfer yield is reduced by 40% on cooling the His83 derivative from 290 to 250 K, with little change on further cooling to 150 K. This finding is consistent with the weak temperature dependence reported for the intramolecular photoinduced charge transfer in the His83 protein below 220 K.^36^ In contrast, the spin polarization for electron transfer in the His83 derivative decreases monotonically from ∼20% at 290 K, to 3% at 200 K, and 0% at 150 K. The two mutants show similar behavior between the temperature dependences of the charge-transfer yield and the spin polarization. Hence, the photoinduced charge-transfer yields are weakly temperature-dependent, but their spin polarizations are strongly suppressed upon lowering the temperature.

#### Spin-Filtering by Azurin

In order to contrast the spin polarization that is found for the photoinduced electron transfer with the intrinsic spin filtering by azurin in the dark, magnetoresistance measurements for films comprising native wild-type azurin (unmodified by Ru(II) bipyridyl) and its temperature dependence were studied in a junction device without illumination. Figure 2A illustrates the four-point probe design that was used to measure the resistance through the junction, and details on the structure are provided in ref (26). A constant tunnelling current of 1 mA was passed from the bottom of an Au electrode through the protein and MgO films to the top Ni/Au electrode. The voltage drop through the film was monitored with the other two electrode contacts. By changing the external magnetic field that was oriented along the direction of the current flow, the magnetic field dependence of the resistance (the magnetoresistance) of the device was measured. Figure 2C shows the magnetoresistance (MR) as a function of the applied magnetic field (*H*) at five different temperatures, where MR (%) is defined as
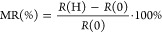
1*R*(*H*) is the resistance at field strength *H* and *R*(0) is the resistance at zero field.

Figure 2B shows that the MR signal has an antisymmetric shape versus field that is typical of CISS-based devices.^26−28,37^ The asymmetric shape of the MR curves, results from the electrons being spin-polarized by the chiral protein and the spin filtering by the Ni layer, which depends on the magnetic field strength and direction. The combination of the two filters (the protein and the Ni), and their different field dependences gives rise to the antisymmetric shape for magnetoresistance curves (Figure 2B). The low MR values are a result of the protein layer having many pinholes that allow current leakage. This current is not spin-polarized and therefore creates a large magnetic independent current on top of which there is a smaller magnetic-dependent current that passes through the protein.

Figure 2C shows the change in the MR signal amplitude as a function of temperature (for 0.8 and −0.8 T fields): the magnitude of the MR drops by almost a factor of 2 on cooling from 300 to 4 K. The decrease of MR as the temperature is reduced, shown in Figure 2C, is much weaker than that found for the spin polarization reported in Figure 1D. It is important to appreciate that while the data in Figures 1D and 2 each indicate the spin filtering property of the protein, the paths of the electrons are different for the two cases. In the case of Figure 1D, the Cu^2+^ ions are necessarily involved in the photoinduced electron transfer process, while in the case of Figure 2 the electrons flow between two electrodes and only part of them are affected by the copper ion. Analyses of conductance data, which compare apo- and Cu-azurin films, show that the transport mechanism through the protein films involves the Cu (II) species;^38,39^ however, other current paths can also contribute. The difference in the spin current pathways in the two experiments is reflected in the activation energies that are obtained from data presented in Figures 1D and 2C, which are about 40 and 13 meV, respectively. Thus, the photoinduced electron transfer pathway accessed in the Ru-modified azurins, which necessarily involves the Cu(II) ion, shows a stronger temperature dependence of the spin filtering than does that of native azurin in the dark.

#### Spin Polarization Accompanies Charge Polarization

Previous studies have shown that the CISS effect correlates with the optical activity of the system through which the electron is transferred and both are related to the anisotropic polarizability of the molecule.^16^ To measure the spin polarization that accompanies charge polarization, we adsorbed the protein on a Hall device (Figures S4 and S5), and the device was put inside an electrolyte solution. A gate electrode was placed on top, electrically insulated from the solution. Upon applying pulses of electric potential between the gate and the device, a Hall potential was measured as shown in Figure S5B. The Hall potential increases linearly with the applied gate voltage, and its sign depends on the sign of the applied field (Figure S5C). The applied gate voltage drives charge reorganization in the protein. Because the charge reorganization is accompanied by spin polarization, it generates a magnetization and a Hall voltage in the device.^18^ This additional test confirms the spin-dependent charge reorganization in azurin.

### Theoretical Models for Spin-Polarized Electron Transfer

Two possible mechanisms were explored in order to rationalize the observed spin-polarized electron transfer and its temperature dependence. The first was based on changes in employed tunneling pathways as a function of temperature, and the second was based on vibronically induced spin polarization.

#### Temperature-Dependent Tunneling Pathways

**Figure 3:**
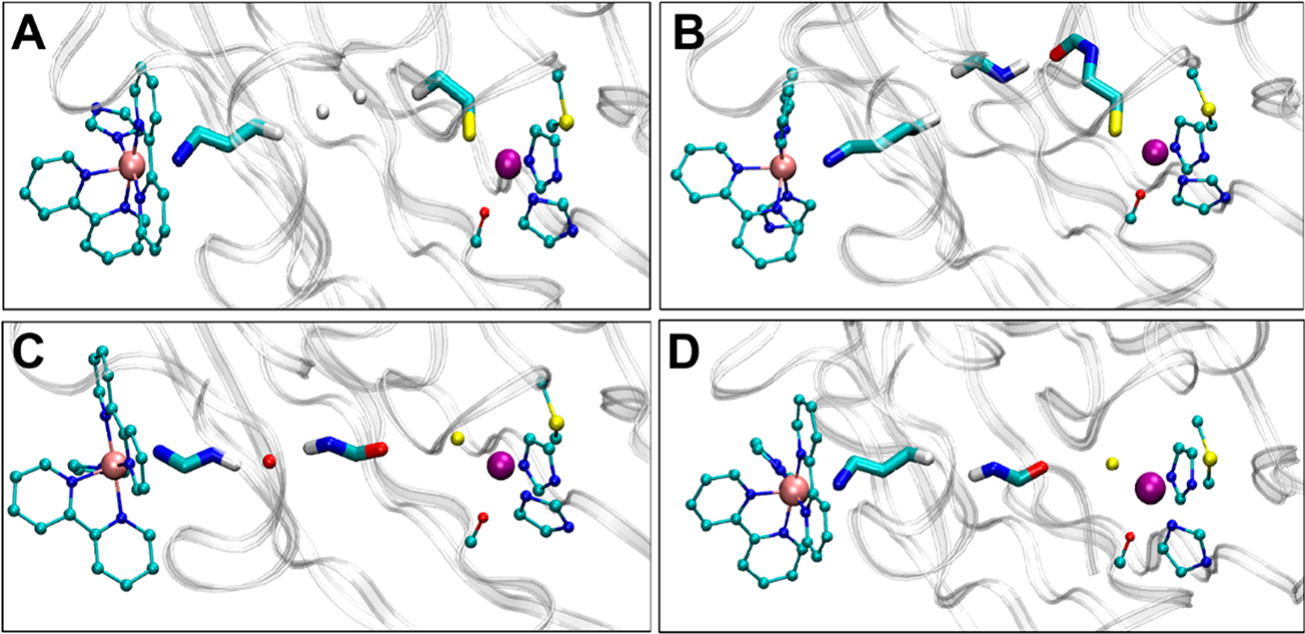
Dominant coupling pathways for His83. (A–D) Examples of the four electron tunneling pathways, which are identified in detail in Table S1, for the His83 protein at 290 K are shown: (A) pathway 1, (B) pathway 2, (C) pathway 3, and (D) pathway 4. The renderings were generated using VMD.^45^

If the dominant electron tunneling pathways change as a function of temperature, then it is possible that a low spin polarization pathway active at low temperature could swap with a high spin polarization pathway operating at elevated temperature. To examine this possibility, the case of Ru-His83 azurin, which exhibits the highest spin polarization, was explored by the electron tunneling pathway analysis^40−43^ using molecular dynamics which sampled protein conformations at 290 and 250 K (see Figures S6 and S7 and the Supporting Information for details). Since the experiments show similar results for charge transfer via the His83 protein at 250 K and lower temperatures, the analysis of the structure–charge-transfer relationship at 250 K, compared to that at 290 K, may also apply to lower temperatures. We first identified the strongest Ru-to-Cu electron tunneling pathway in His83 azurin for each selected MD snapshot and computed the occurrences of the strongest tunneling pathways in the MD sampled snapshots at 250 and 290 K (Table S1, Figure 3).

The two strongest electron tunneling pathways that occur with the highest frequencies were unchanged in the structure at 290 and 250 K (pathways 1 and 2 in Table S1). However, their overall occurrence frequency was larger at the lower temperature. That is, these are the two strongest pathways in ∼61 and 80% of the MD snapshots at 290 and 250 K, respectively. At 290 K, we found a more frequent occurrence of two tunneling pathways through Asn47 that are assisted by an internal water molecule (similar to mediation found in other proteins)^44^ and proceed from Ru to Cu following an almost straight line (Figure 3C), corresponding to a minimal donor-to-acceptor distance and relatively large donor–acceptor coupling. Although the relative contributions of these pathways changed somewhat with temperature (from 290 to 250 K), their relatively small overall contribution, as compared to those of pathways 1 and 2, make them unlikely to contribute much to a change in the observed spin polarization.

We found a slightly shorter Ru–Cu average distance appearing at the higher temperature (Table S2), which correlated with a larger occurrence probability of the direct pathways 3 and 4. In fact, averaging the Ru–Cu distance over the snapshots for which pathway 2 (i.e., the most meandering among the stronger tunneling pathways shown in Figure 3) contributed most strongly to the coupling, the Ru–Cu distances at 290 and 250 K differed by less than 0.1 Å, while the distance difference increased to about 0.4 Å when the averages were taken over the MD snapshots where pathways 3 and 4 dominate (Table S2). Comparing the protein system at two temperatures, we see that the slight compression, enabled by the enhanced protein flexibility at higher temperature, allows a more frequent occurrence of efficient tunneling pathways along the donor–acceptor direction, partly involving internal water molecules.

On average, we found that Cu(II) is slightly out of the equatorial plane (defined by the two His imidazolyl ligands and the Cys thiolate) on the side of the Met ligand based on the MD sampled structures. The average Cu(II) distance from the equatorial ligand plane is larger at 250 K (0.045 Å) than at 290 K (0.037 Å). At 250 K, however, the methionine position moved, so the coordination bond between Cu and the Met 121 sulfur is more extended than that at 290 K (0.35 vs 0.36 nm; see Table S2 and Figure S8). This stretching may correlate with the increased occurrence of the strongest tunneling pathways through Cys 112 (Table S3) and the adjacent Phe 111.

The reduced average Cu–Ru distance and the related larger contribution from more direct tunneling pathways produced a stronger electronic coupling between Cu and Ru at 290 K than that at 250 K for the His83 derivative, in agreement with the data in Figure 1C. The ratio between the electron transfer rates at the two temperatures (as determined by the ratio of the mean-square electron tunneling pathway decay products, Table S4) was within the error bars of Figure 1C. A closer agreement to the experiments is achieved by considering a kinetic model of the protein-electrode system that includes the effects of the electron transfer from Cu(I) to the Ag film. In fact, through a more detailed modeling (see the Supporting Information and Tables S5 and S6), we found that the probabilities that charge accumulation on Ag (which is proportional to the measured voltage in Figure 1C) at 290 and 250 K is in the ratio 1.8, in good agreement with the experimental results (see the data points for His83 in Figure 1C).

MD simulations and pathway analysis for His83-modified azurin indicate that gross changes in the electronic coupling pathways are not responsible for the strongly temperature-dependent spin polarization. The molecular dynamics and DFT analysis indicate that the average structure of the protein has the Cu(II) ion out of plane. This finding is consistent with the crystal structure and Raman data^46^ near room temperature. The increase in the Cu to Met distance on cooling from 290 to 250 K does not affect the charge transfer as the dominant coupling pathways proceed through the copper’s cysteine ligand.

#### Vibronically Activated Spin Polarization

The coupling of the Cu(II) coordination site to protein vibrations has been observed before by electron paramagnetic resonance (EPR) measurements.^47,48^ The dependence of the EPR spectra on temperature indicate a more isotropic *g*-tensor as temperature increases. It was concluded that at room temperature the spectral changes are due to vibronic coupling of Cu(II) and the methionine ligand (i.e., vibrational distortions along the low frequency (177 cm^–1^) Cu–Met coordinate mixes excited states of the Cu(II) d electrons with the ground state).^28,29,49^ At low temperature, this vibronic coupling is reduced significantly.

**Figure 4:**
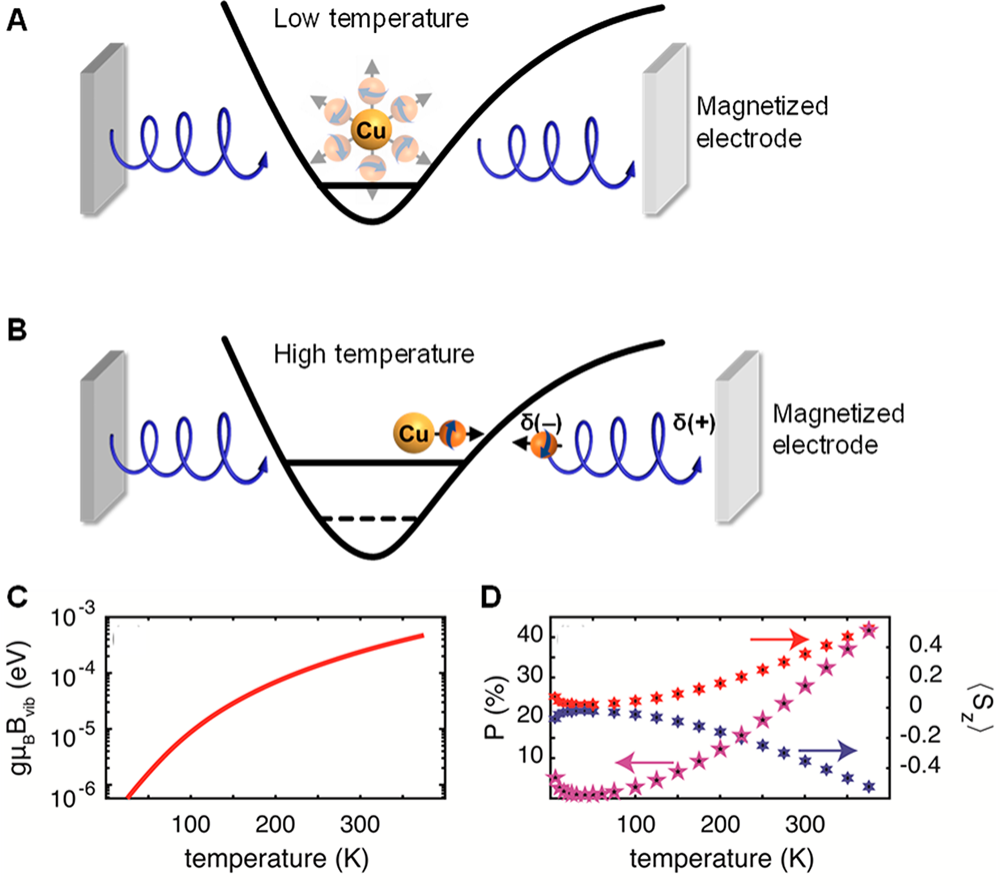
Vibronically activated spin polarization model. (A, B) Scheme of the Cu(II) ligand complex model. Here two chiral molecules are interacting with the Cu(II) ion which is located in anharmonic potential. The chiral molecules serve as a bridge for electron conduction between a ferromagnetic and normal metallic lead (the blue and light gray squares, respectively). (A) Diagram representing low-temperature conditions under which the Cu(II) is located near the bottom of the potential and its surrounding is isotropic. (B) Diagram representing the high-temperature situation, in which the Cu(II) is located (on average) away from the harmonic part of the potential and its environment is anisotropic. (C) Vibrationally induced magnetic field, **B**_vib_, as a function of temperature. (D) Ionic spin moment (right axis) for external field *B* = 0.35 T (red) and *B* = −0.35 T (blue), and chiral-induced spin polarization, *P* (left axis), as a function of temperature.

We apply here a model that describes vibrational effects on chiral-induced spin selectivity.^50^ To simplify, we consider a model that assumes electron transport through a molecular junction located between two electrodes, and we approximate the molecule by a copper ion located between two anharmonic chiral oscillators. Figure 4A,B shows a schematic illustration of this simplified model system. This model is intended to account for the vibronic coupling between the Cu(II) ion and its coordination sphere of amino acid residues of the azurin’s peptide chain. The electron-vibrational coupling leads to an indirect exchange between the Cu(II) and its ligand shell, which manifests as an effective magnetic field **B**_vib_ acting on the Cu(II). The vibronic coordinate which couples the Cu(II) to the ligand sphere is modeled as anharmonic, and this feature is represented in Figure 4 by the asymmetric (Morse-like) potential energy curve.

Figure 4A,B shows the diagrams for both low and high temperatures. The copper–ligand vibrational motion is more harmonic at lower temperatures than at elevated ones. As the Cu(I/II)–ligand vibration(s) proceeds, it generates charge displacements in the chiral ligand’s electron cloud, and these are accompanied by partial spin polarization of the electron cloud.^51^ Because the amplitude of the motion is small at low temperature and is nearly harmonic, the local fluctuating **B**_vib_ field is small. At higher temperatures (Figure 4B), larger displacements occur along the vibrational coordinate, and they access more anharmonic regions of the vibrational coordinate between the copper and its chiral ligands, giving rise to a net local magnetic polarization.^51^ The temperature dependence of azurin’s EPR spectrum has been measured (see refs (47−49)) and supports this model. While the *g*_⊥_ component of the *g*-tensor does not change significantly with temperature, its *g*_∥_ component changes significantly above 50 K, with an activation energy of about 160 cm^–1^. The vibronically driven exchange coupling along the *g*_∥_ direction generates a change in the magnetic moment with temperature that spin polarizes the copper–ligand environment, increasing the root-mean-square magnetic moment of the copper ion (see eqs S4b and S5).

The temperature dependence of the Cu(II) site’s magnetic moment (spin polarization) can explain the temperature dependence of the observed protein’s spin filtering. At low temperature, the electron transmission through Cu(II) is spin independent and **B**_vib_ is small. Upon increasing the temperature, **B**_vib_ increases, and the copper’s spin polarization increases, becoming oriented in the protein’s molecular frame. Hence, the transmission of electrons will depend on their spin direction relative to that of the Cu’s spin polarization, making the electron transport spin-selective. Because the electron spin polarization on the Cu(II) increases with temperature, the spin filtering of the incident electron flux through the molecular junction is higher.

On the basis of the model described above, we simulated the charge current that flows through the Cu(II) coordination sphere between 5 and 400 K. The parameters used in the simulation are given in the Supporting Information. The plots in Figures 4 C,4D show the induced effective magnetic field, **B**_vib_, the ionic spin moment, and chiral induced spin polarization, computed as a function of temperature. Using the parameters reported in the “Methods” section, **B**_vib_ grows 100-fold as the temperature increases from 50 K to room temperature (Figure 4C), and it produces a significant spin moment (Figure 4D, right axis). The charge current *I* flowing through the system can be written as
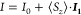
2where *I*_0_ is the electron flux that is not through the copper and ***I***_**1**_ represents the flux of electrons through the copper. Figure 4D shows a plot of the chiral induced spin polarization, defined as *P* = 100%·|⟨*S*_*z*_⟩·***I***_**1**_/*I*_0_|. The value of the spin polarization in this analysis is similar to the experimental value.

## Conclusions

Photoinduced electron transfer reactions of Ru-modified azurins are very weakly dependent on temperature, quite unlike the spin polarization of electron transfer, which exhibits strong dependence. The weak dependence of charge transfer on temperature is consistent with previously reported photoinduced intramolecular electron transfer rates for these Ru-modified azurins.^3^ The earlier findings are in accord with the above coupling pathway analysis for His83 azurin, which indicates that the donor–acceptor squared coupling decreases by less than 2-fold from 290 to 250 K. The weak temperature dependence of the charge-transfer yields shown in Figure 1C for the azurin assemblies on Ag films suggests that the activation free energies associated with carrier injection to the electrodes is also small. In contrast, the spin polarization response depends strongly on temperature.

Spin polarization has been measured previously in cytochrome c,^18^ bacteriorhodopsin,^19^ and photosystem I.^52^ While spin-dependent transport was detected in those systems, temperature dependences were difficult to extract because of strong temperature dependences of the charge-transfer processes. Here we have the advantage of measuring temperature-dependent spin polarization in Ru-azurins where the underlying charge-transfer kinetics depend weakly on temperature. This approach has allowed us to probe the temperature-dependent spin polarization separately from the temperature dependence of charge-transfer reactions. In so doing, as spin filtering is thermally activated, we infer that it arises from vibronically activated electronic exchange interactions at the copper coordination site(s).

The model used here to describe the temperature-dependent spin polarization of azurin invokes vibrationally induced exchange interactions between the chiral ligands of the coordination sphere and the copper ion. As the temperature increases, the amplitude of the displacement along the anharmonic metal–ligand coordinate grows, and the copper ion interacts with one side of the coordination sphere more than the other, which polarizes the copper spin. As the Cu(II)-to-ligand distance decreases, the chiral ligand becomes both charge-polarized and spin-polarized.^17^ The unpaired electron on Cu(II) interacts with spin-polarized electrons on the ligand via spin-exchange. If the exchange interaction is antiferromagnetic, then the electron(s) on Cu(II) align antiparallel to the spin polarization of the ligand. As a result, electrons transfer from the copper site more readily if the electron’s spin is oriented antiparallel to that of the copper (Figure 4).

In conclusion, studies of charge and spin transfer for Ru-modified azurin films reveal very different temperature dependences, with the charge transfer being essentially temperature-independent and the spin transfer increasing with temperature (*E*_act_ is 10s of meV). Because the spin polarization increases with temperature, it seems likely that spin-polarized electron transfer may be ubiquitous in living systems at ambient temperatures. The benefit of spin-polarized electrons for enhancing chemical selectivity in multielectron transfer processes is known^53^ and is likely manifested in biochemical cycles and cascades. In addition, the spin polarization reduces backscattering of the electrons within the protein, due to the coupling of the electron’s linear momentum to its spin.^17^

## Supporting Information Available

Materials; preparation and measurement of MTJ device and Hall device; XPS and absorption spectroscopy data; theoretical study details (PDF)
