# Supplementary material for: Temperature Dependence of Charge and Spin Transfer in Azurin
Source: J Phys Chem C Nanomater Interfaces. 2021 Apr 29;125(18):9875–83. doi: 10.1021/acs.jpcc.1c01218 (PMC8154855; doi:10.1021/acs.jpcc.1c01218)
Supplement: Supplementary file 1 — jp1c01218_si_001.pdf [file jp1c01218_si_001.pdf]

Supplementary Information for

Temperature Dependence of Charge and Spin Transfer in Azurin

*Yutao Sang[a], Suryakant Mishra[a], Francesco Tassinari[a], Karuppannan S. Kumar[a], Raanan Carmieli[b], Ruijie D. Teo[c], Agostino Migliore[c,d], David N. Beratan[c], Harry B. Gray[e], Israel Pecht[f], Jonas Fransson[g], David H. Waldeck[h], Ron Naaman[a,]**

[a] Department of Chemical and Biological Physics, Weizmann Institute, Rehovot 76100, Israel
[b] Department of Chemical Research Support, Weizmann Institute of Science, Rehovot 76100, Israel
[c] Department of Chemistry, Duke University, Durham, North Carolina 27708, United States
[d] Department of Chemical Sciences, University of Padova, Via Marzolo 1, Padova 35122, Italy
[e] Beckman Institute, California Institute of Technology, Pasadena, CA 91125
[f] Department of Immunology, Weizmann Institute, Rehovot 76100, Israel
[g] Department of Physics and Astronomy, Uppsala University, Uppsala 752 36, Sweden
[h] Department of Chemistry, University of Pittsburgh, Pittsburgh PA 15260; orcid.org/0000-0003-2982-0929

* Corresponding author: Ron Naaman
Email: ron.naaman@weizmann.ac.il

This PDF file includes:

Experimental details
Figures S1 to S5
Theoretical study
Figures S6 to S9
Tables S1 to S6
SI References

1. Experimental details

1.1 Materials.

The silicon wafers used as substrates were acquired from Virginia Semiconductor (crystal orientation <100>, thickness 525±251 μm). The water used in the experiments was purified using a Millipore Synergy® Water Purification System (18.2 MΩ/cm at 25°C). Other chemicals were purchased from Merck, Gadot, or Bio-Lab (A.R/HPLC grade).

1.2 Preparation of magnetic tunnel junction (MTJ) device.

The device (Ni/AlOx/Ag) was prepared by photolithography followed by e-beam evaporation on silicon substrate (<100>, 400 Ω per cm^2). A 150 nm thick Ni line (1 μm wide, 1 mm long) was evaporated on a 5 nm Ti adhesion layer. 0.5 nm AlOx layer was then deposited by atomic layer deposition (Fiji F200, Cambridge Nanotech.). The top 50 nm thick perpendicular Ag line was evaporated without any adhesion layer. Gold contacts for wire-bonding are 150 nm thick. Monolayer formation and device measurements are presented in the main text.

1.3 X-ray Photoelectron Spectroscopy (XPS).

XPS measurements were carried out with a Kratos AXIS ULTRA system, using a monochromatic Al (Kα) X-ray source (hν = 1486.6 eV) at 75 W and detection pass energies between 10 and 80 eV with takeoff angle of 0° and 45°.

1.4 Absorption spectroscopy.

The UV-Vis absorption measurements were taken with a Cary 5000 UV-Vis-NIR spectrophotometer.

1.5 Preparation and measurement of Hall device.

AlGaN/GaN wafers on a sapphire substrate (purchased from NTT) were used for the fabrication of the Hall devices. The wafer is composed of a nucleation layer, and an intrinsic-GaN layer of 1800 nm thickness. On top of the intrinsic-GaN layer, 20 nm of an intrinsic-AlGaN layer was present. Finally, a capping layer of 2 nm thickness of GaN was present on intrinsic-AlGaN (**Fig. S4**). All the Hall devices were prepared by standard photolithography in a class 1000 clean room. To prepare ohmic contacts, a metallic multilayer i.e., Ti (20 nm)/Al (100nm)/Ni (40 nm)/Au (40 nm) was annealed at 850°C. A size of 500 μm in length and 40 μm in width of the active area of the channel was also coated with 2 nm Ti and 5nm Au for molecular adsorption (**Fig. S5**).

The device surface was first cleaned by boiling in acetone and ethanol (10 min each), then cleaned by a 5 min ozone treatment (UVOCS), and was subsequently immersed in ethanol. After 30 min, the device was dried with N_2 stream and azurin solution (1 mg/mL in buffer, pH=7) was immediately drop-cast on to the surface and stored in a sealed humidity box at room temperature for 4 hours. After incubation, the device was gently rinsed with DI water and dried with N_2 stream.

After growing the monolayers of the azurin monolayer on the channel, the device chip was glued to a chip holder using double-sided tape. The pads of the electrodes were connected to a chip holder using wire binding. All the connections were passivated by using a high-quality RTV silicone glue to avoid charge leakage. To maintain the aqueous environment during the measurements, we prepared a polydimethylsiloxane (PDMS) cell with a 200 μL capacity that was glued on top of the device on the chip holder.

A glass coverslip coated with 100 nm gold was used as the gate electrode, and was placed on the top of PDMS cell that contained a 0.1 M HEPES buffer solution. A constant potential pulse of different magnitudes was applied to this coverslip, providing the electric field that polarized the monolayer. During the experiment, a constant current of 10 μA was maintained between the source (S) and drain (D) electrodes. The varying gate voltages and the constant S-D current were applied using a dual channel Keithley 2636A source measuring unit. The Hall voltage was recorded using a Keithley 2182A nanovoltmeter, and it was measured while constant current was maintained in both directions, i.e., from S to D and D to S, to correct for the error caused by the asymmetry of the Hall device. All measurements were performed in a dark Faraday cage.

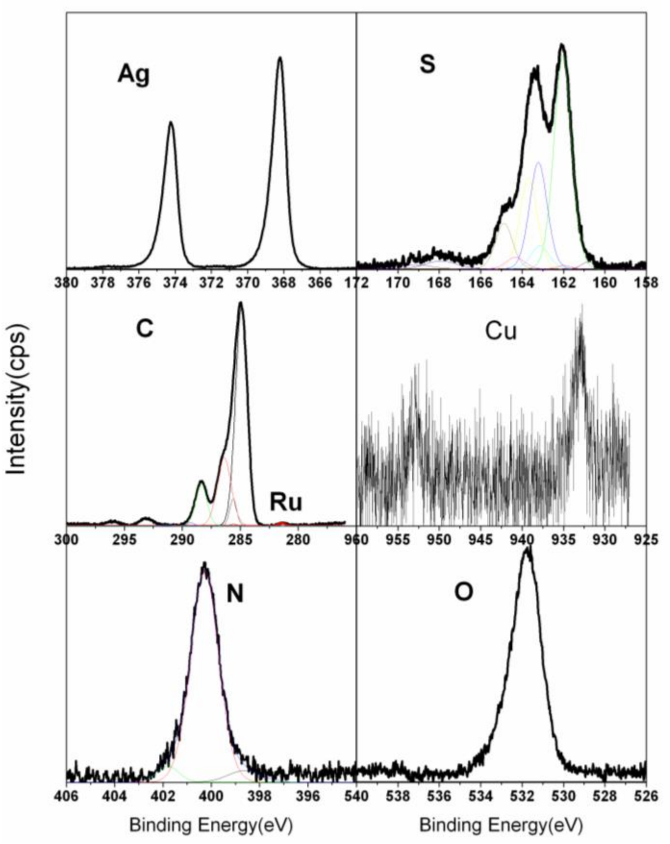

Fig. S1. XPS of monolayer of His 83 azurin on silver surface.

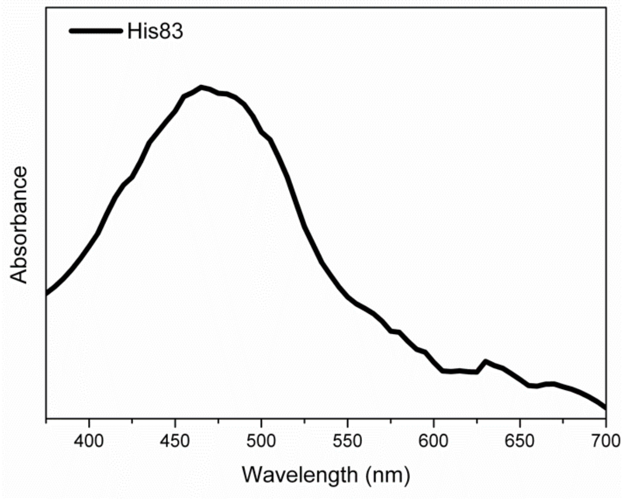

Fig. S2. Absorption spectrum of Ru(II)(bpy)2(im) His 83 monolayer on 50 nm of Ag film.

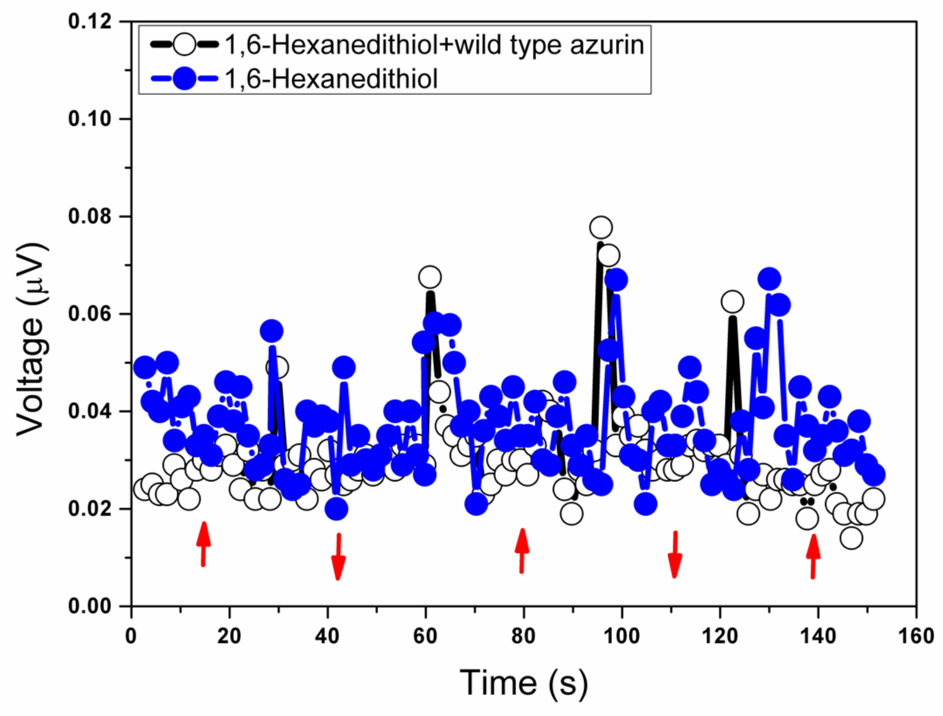

Fig. S3. Time profiles of the voltage between silver and nickel layers for magnetic fields pointing towards or away from the organic layer (red arrows), as measured at 290 K for silver coated with a monolayer of 1,6-hexandithiol and wild type azurin illuminated by 532 nm.

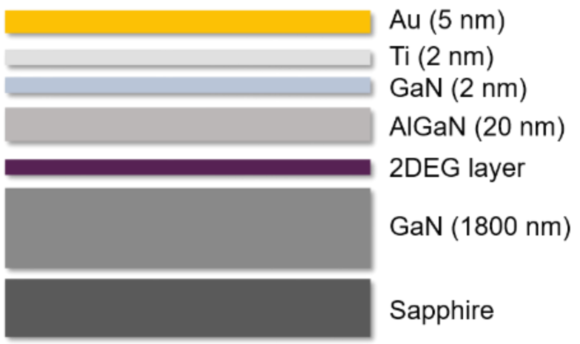

Fig. S4. Composition of the Hall device channel.

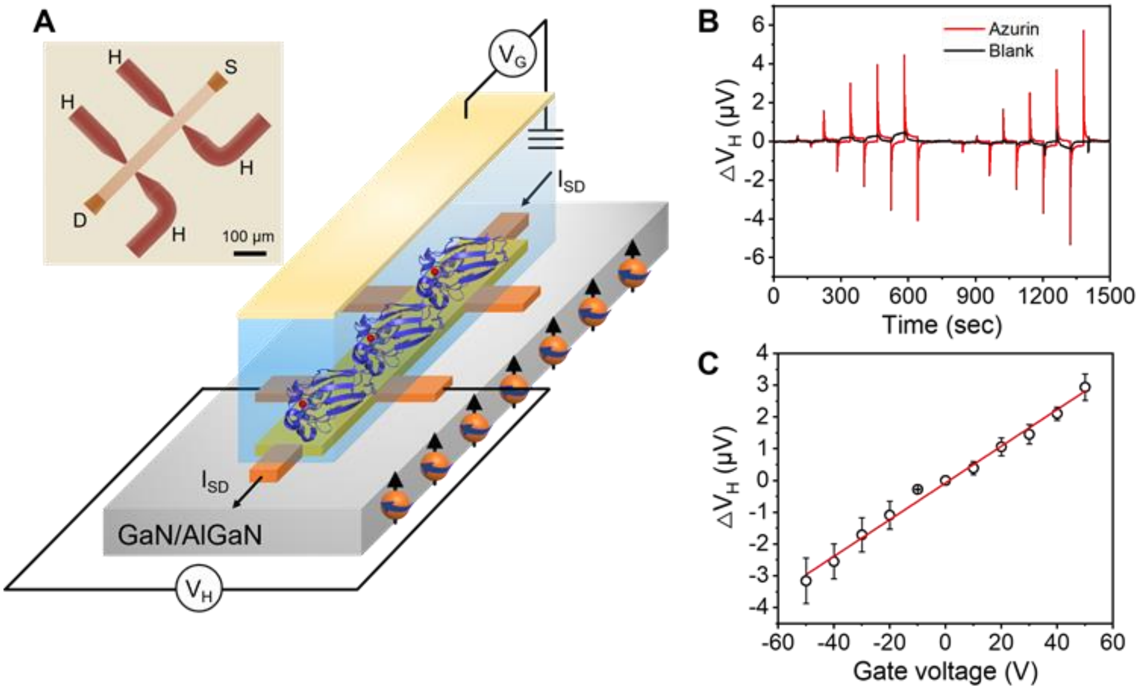

Fig. S5. Electric field induced spin polarization in azurin measured by the Hall effect. (*A*) Scheme of the Hall-based setup for the spin-dependent polarization studies, where the device made from GaN/AlGaN is coated with a monolayer of wild-type native azurin. A gate electrode, insulated from the solution, is used to apply an electric field. Inset shows the optical microscopic image of the Hall device patterned on the substrate. A current of 10 μA is derived between the source and drain electrodes. (*B*) The Hall potential measured with sequential gate pulses from +50V to −50 V in steps of 10 V. (*C*) The Hall potential as a function of the applied gate voltage.

For establishing the spin dependent electron transport through the azurin under conditions that are as close as possible to the physiological one, we adsorbed the protein on the devices shown in **Fig. S5A**. These Hall devices are made of GaN/AlGaN structure with a two-dimensional electron gas (2DEG) interface layer. Notice that the device applied here is different from the common Hall device which is applied using an external magnetic field acting perpendicular to the surface of the device. As a result of the Lorentz force, electric potential can be measured perpendicular to the source-drain current and to the magnetic field. This potential is the “Hall potential”. In the device used here, no external magnetic field is applied, but instead the protein is adsorbed on the surface of the device. In the experiment, the azurin is adsorbed on the device which is placed within the electrolytic solution using a PDMS cell. A top gold electrode is placed on the top of the solution facing

the device and it is used to apply the electric field. On the application of the electric field, the protein is charge polarized and if this charge polarization is accompanied by spin polarization, the spins that are injected from the protein into the device create a magnetic field which can be monitored by the Hall probes (**Fig. S5B**).

2. Theoretical study details

2.1 MD simulations.

The azurin coordinates were obtained from the PDB file with ID 1JZE. The MD simulations at the 290 K and 250 K temperatures were carried out using the NAMD 2.11 program.[1] Both the 290 K and 250 K systems were solvated with a water box extending 10 Å on each side of the protein. The resulting unit cell vectors were (in Å): (**65.000**, **61.000**, **60.000**).

H-O and H-H distances in the water molecules were constrained using the SHAKE algorithm.[2] We used the particle mesh Ewald summation method[3] (1Å grid spacing) to calculate the electrostatic potentials. Full electrostatic energy evaluations were performed every two time steps. The 1-4 electrostatic interactions used a scaling factor of 0.833333. The cutoff distance for van der Waals interactions was 12 Å. Atomic pairs within 14 Å were considered for periodical interaction energy calculation.

Each system was minimized for 80000 steps. The energy minimization was followed by solvent equilibration (using Langevin dynamics with a damping coefficient of 1.0 ps^{-1}) with fixed solute atoms at 293 K (original crystallization temperature of 1JZE[4]) for 150 ps. The positions of solute atoms were then released for the next 75 ps of equilibration. This equilibration was followed by further 50 ps of equilibration at 290 K for the first system. The second system was equilibrated further 50 ps at each of the following temperatures in the order: 280 K, 270 K, 260 K, and 250 K. Next, 0.5 ns of NPT dynamics with Nosé-Hoover Langevin piston pressure control was carried out for each system (pressure = 1 atm, barostat oscillation period = 100 fs, damping time scale = 50 fs, damping coefficient = 2.0 ps^{-1}).

The MD production run lasted 100 ns and 110 ns for the 290 K and 250 K systems, respectively, with a time step of 0.5 fs. The RMSDs of the 290 K and 250 K systems spanned ranges of about 1.27 Å and 0.65 Å, respectively (**Fig. S6** and **S7**). We extracted the MD snapshots every 0.1 ns from the time windows 20-100 ns at 290 K and 20-108 ns at 250 K.

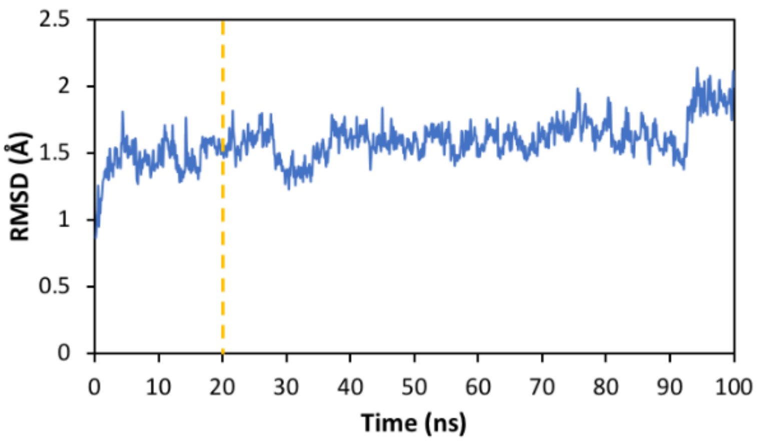

Fig. S6. RMSD (without H atoms) along the MD production run for the Ru-modified azurin at 290 K. The MD snapshots for pathway analysis were selected after 20 ns (yellow dashed line).

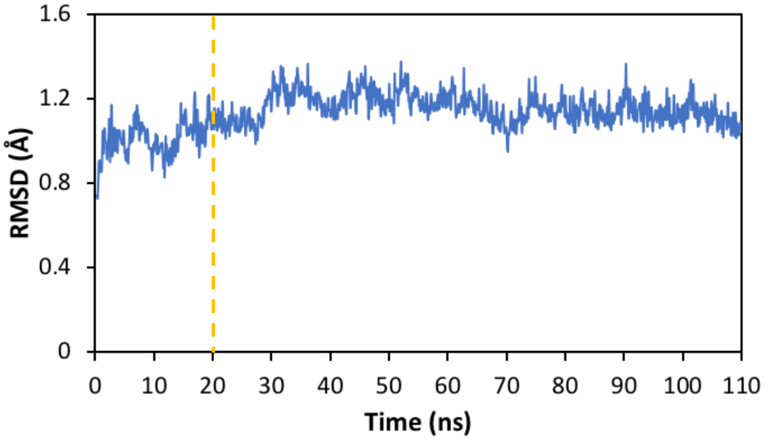

Fig. S7. RMSD (without H atoms) along the MD production run for the Ru-modified azurin at 250 K.

2.2 Computational details about the ET pathway analysis.

The structure and electron transfer properties of the selected MD snapshots were analyzed using the Pathways 1.2 plugin [5] for VMD.[6] The 'strength' of the electron tunneling pathways is measured by the electronic coupling decay factor (or decay product)[7,8,9,10,11]

$$U_{DA} = \prod_i \varepsilon_c(i) \prod_j \varepsilon_H(j) \prod_k \varepsilon_S(k)$$

where i, j, and k distinguish the through-covalent bond, through-hydrogen bond, and through-space tunneling steps between the electron donor and acceptor in a given electronic tunneling pathway. $\varepsilon_c = 0.6$, $\varepsilon_H = \varepsilon_C^2\, e^{-\beta_S(R_H - 2.8)}$ (R_H is the length of the hydrogen bond), $\varepsilon_S = \varepsilon_C\, e^{-\beta_S(R_S - 1.4)}$ (R_S is the length of the through-space step), and β_S = 1.15 $Å^{-1}$.[5,12] Including the H atoms explicitly in the pathway analysis, we observed that the combination of a through-bond step (from the electron donor to the hydrogen atom) and a through-space step (from the hydrogen to the electron acceptor) corresponded to a smaller decay factor (and therefore a larger coupling factor) than the through-H bond step. Therefore, the pathway program described the H bonds as through-bond steps followed by through-space steps.

2.3 Structural parameters, coupling pathways, and charge transfer modeling.

We started our theoretical analysis with a geometry optimization of the Cu site, followed by a single-point electronic structure calculation. In the optimized geometry, Cu is out of plane on the side of the glycine axial ligand (Gly45) by 0.067 Å. The highest occupied molecular orbital (which is singly occupied) is mainly localized on the p_x and p_z atomic orbitals of the methionine (Met121) ligand's sulfur atom, while the lowest unoccupied molecular orbital is distributed on the Cu and the p_y of the cysteine (Cys112) ligand's sulfur atom. Cys112 (which has been focus of mutation studies because its bonding interactions influence azurin spectroscopic properties[13,14]) participates in the dominant Ru-to-Cu electron tunneling pathways (see **Tables S1, S3**). **Fig. S8** shows the average structures of the Cu site based on the MD simulations at 290 K and 250 K. **Fig. S9** shows the distributions of the Cu-Ru distances against the Cu-Met distances in the two simulations. Further information on distances relevant to the Cu-Ru electron transfer are reported in **Table S2**.

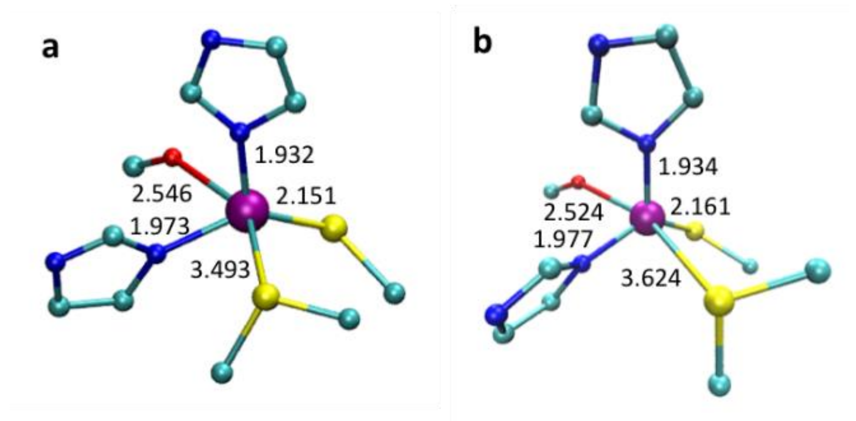

Fig. S8. Average bond distances (in Å) between the ligands (Gly 45, His 46, Cys 112, His 117, Met 121) and the Cu center over the selected MD snapshots, at the temperatures of (a) 290 K and (b) 250 K. The average coordination-bond lengths correlate with previous crystallographic data taken at 293 K.[15] The Cu-S_D average distance obtained from the MD simulation at 290 K is a little larger than the crystallographic one. However, apart from the limits of comparing crystallographic and MD structures, this distance decreases with increasing temperature from 250 K to 290 K, and a possible further (nonlinear) decrease may occur near room temperature. The figure was generated using VMD.[6]

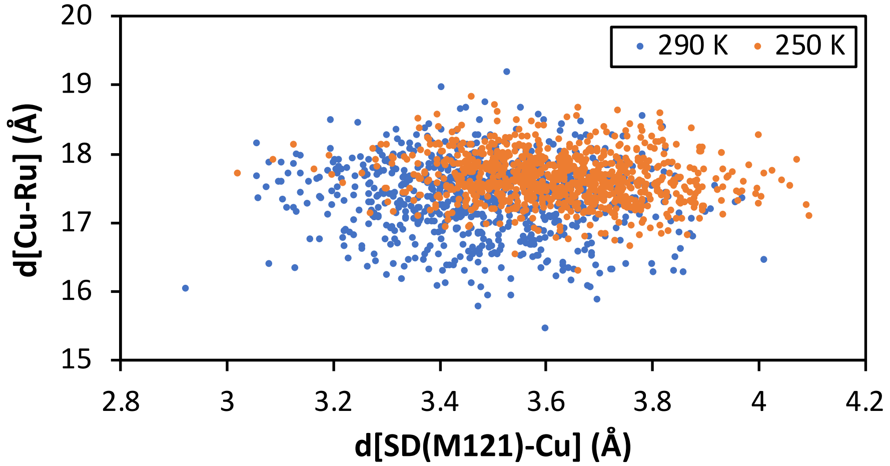

Fig. S9. Cu-Ru distance versus the distance between Cu and the sulfur of the M121 ligand (S_D). The diagram shows no correlation between these two distances. The distributions of the Cu-S_D and Cu-Ru distances are shifted towards larger values at the lower temperature.

Table S1. Frequency of occurrence of the strongest tunneling pathways between Ru^{III} and Cu^{II} (electron donor-to-acceptor transition) across the His83 protein dynamic evolution at the two indicated temperatures. The pathways involving the same amino acid residue sequence are marked with the same color. The listed tunneling pathways cover more than 90% of the system snapshots selected. One-letter codes are used for the amino acid residues.

	290 K	%	250 K	%
1	Ru-H83-V49-C112-Cu	46.5	Ru-H83-V49-C112-Cu	55.8
2	Ru-H83-V49-F111-C112-Cu	14.6	Ru-H83-V49-F111-C112-Cu	24.2
3	Ru-H83-WAT-N47-C112-Cu	11.9	Ru-H83-W48-V49-F111-C112-Cu	8.8
4	Ru-H83-N47-C112-Cu	8.6	Ru-H83-N47-C112-Cu	3.1
5	Ru-W48-N47-C112-Cu	4.0	Ru-H83-WAT-N47-C112-Cu	2.6
6	Ru-H83-W48-V49-F111-C112-Cu	3.9	Ru-H83-W48-N47-C112-Cu	1.8
7	Ru-H83-T84-N47-C112-Cu	3.6	Ru-N47-H46-G45-Cu	1.1

Table S2. Average distances between the indicated atoms over the MD snapshots corresponding to the indicated dominant electron tunneling pathways (namely, all pathways, the 'tortuous' path 2, and the more direct pathways 3 and 4, as ordered for the H83 protein at 290 K). We obtain very similar values for the closest distance between C112 and N47 when we average this distance over the MD snapshots corresponding to tunneling pathway 2 or to pathways 3 and 4, and no significant difference is obtained at the two temperatures. Thus, we argue that the wide protein motion leading to the more frequent occurrence of pathways 3 and 4 (as well as a smaller Cu-Ru average distance) at 290 K than at 250 K does not affect appreciably the local relative arrangement of C112 and N47. The different pathway-related averages of the Cu site geometry at 290 K produce similar distances.

**Atomic pair (Å), pathway(s)**	**290 K**	**250 K**
Cu-Ru, all	17.355	17.624
Cu-Ru, 2	17.618	17.703
Cu-Ru, 3+4	17.255	
OD1(N47)-SG(C112), 2	3.559	3.568
OD1(N47)-SG(C112), 3+4	3.554	
O(G45)-Cu, all	2.546	2.524
O(G45)-Cu, 2	2.568	2.511
O(G45)-Cu, 3+4	2.561	
ND1(H46)-Cu, all	1.973	1.977
ND1(H46)-Cu, 2	1.970	1.979
ND1(H46)-Cu, 3+4	1.975	
SG(C112)-Cu, all	2.151	2.161
SG(C112)-Cu, 2	2.149	2.162
SG(C112)-Cu, 3+4	2.152	
ND1(H117)-Cu, all	1.932	1.934
ND1(H117)-Cu, 2	1.927	1.937
ND1(H117)-Cu, 3+4	1.933	
SD(M121)-Cu, all	3.493	3.624
SD(M121)-Cu, 2	3.482	3.619
SD(M121)-Cu, 3+4	3.500	

Table S3. Percentages of dominant tunneling pathways involved in the electron tunneling between Ru^{III} and Cu^{II} ($D \rightarrow A$ transition) that pass through each ligand to Cu.

	C112	G45	H46	H117	M121
290 K	94.6	2.6	2.8	0	0
250 K	96.7	1.7	1.6	0	0

Table S4. Mean-square electronic coupling decay factor (decay product) $\langle T^2 \rangle$, in (dimensionless) units of 10^{-10}, for the $D \rightarrow A$ transition. We also present the averages taken over the MD snapshots for which the strongest tunneling pathway passes through the C112, G45 or H46 ligand to Cu. The values in the last two columns are the averages over the MD snapshots with (w) and without (nw) water in the strongest ET pathway. 14.5% (2.8%) of the MD snapshots involves internal water at 290 (250) K.

	$\langle T^2 \rangle$	$\langle T_{C112}^2 \rangle$	$\langle T_{G45}^2 \rangle$	$\langle T_{H46}^2 \rangle$	$\langle T_w^2 \rangle$	$\langle T_{nw}^2 \rangle$
290 K	16.12	16.24	14.73	13.39	22.53	15.13
250 K	12.56	12.68	10.15	7.67	13.85	12.52

To understand the charge flow through the system, we used the kinetic model shown in the scheme below. k_{01} (k_{10}) denotes the effective rate constant for protein excitation (de-excitation). k_{DA} describes the intra-protein electron transfer from the photoexcited Ru site (electron donor, D) to the copper site (electron acceptor, A), while k_{AD} is the rate constant for the converse process. k_{MD} is the rate constant of the downhill interfacial electron transfer from the Ag electrode to the Ru center. The rate constant associated with the converse, energetically uphill transition is neglected (this is a very good approximation in the absence of sufficiently large external bias,[16,17] and it does not affect our conclusions). k_{AM} describes the Cu-to-Ag electron transfer that restores the initial Ru^{II}/Cu^{II} state, while the converse process is neglected.

Reaction scheme

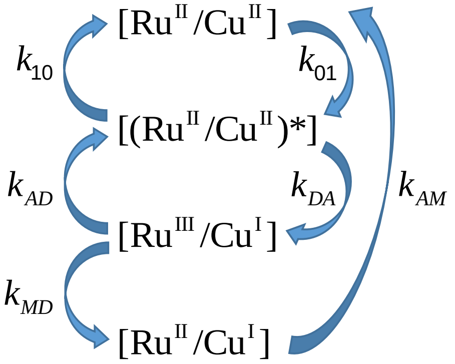

For a given spin-polarization, and under steady-state conditions, the probabilities that the protein complex is in the states of scheme 1 are obtained from the classical-type master equation

$$\begin{cases} \frac{dP_0}{dt} = -P_0 k_{01} + P_1 k_{10} + P k_{AM} = 0 \\ \frac{dP_1}{dt} = -P_1(k_{10} + k_{DA}) + P_2 k_{AD} + P_0 k_{01} = 0 \\ \frac{dP_2}{dt} = -P_2(k_{AD} + k_{MD}) + P_1 k_{DA} = 0 \\ \frac{dP}{dt} = -P k_{AM} + P_2 k_{MD} = 0 \\ P_0 + P_1 + P_2 + P = 1 \end{cases} \quad \textbf{(S1)}$$

In **Equation S1**, P_0, P_1, P_2, and P are the occupation probabilities of the Ru^{II}/Cu^{II}, $(Ru^{II}/Cu^{II})^*$, Ru^{III}/Cu^{I}, and Ru^{II}/Cu^{I} states, respectively, which are proportional to the corresponding concentrations in the protein layer. Ru^{II}/Cu^{I} is the only species that contains an extra electron provided by the metal. If no appreciable electron tunneling between the Ag and Ni electrodes can occur with one of the two spin-polarizations (e.g., spin up, ↑), P also is the probability that one electron hole accumulates on Ag. Then, the Ag-Ni potential difference is proportional to P, which is obtained from **Equation S1** as

$$P = \frac{k_{01}k_{DA}k_{MD}}{k_{01}k_{DA}k_{MD} + k_{01}k_{DA}k_{AM} + k_{01}k_{AD}k_{AM} + k_{01}k_{MD}k_{AM} + k_{10}k_{AD}k_{AM} + k_{10}k_{MD}k_{AM} + k_{DA}k_{MD}k_{AM}} \quad \textbf{(S2)}$$

We make the reasonable assumption that k_{01} is much smaller than k_{10}. Moreover, considering the protein linkage to the electrode, and the proximity of the Ru center to the Ag electrode in particular, we assume that the downhill $M \rightarrow D$ process is much faster than the uphill $A \rightarrow D$ process. The converse is certainly ruled out by the experimental evidence. In fact, for $k_{AD} > k_{MD}$, the probability P in **Equation S2**, and hence the measured voltage, would depend on k_{AD}/k_{DA} (where the $D \rightarrow A$ transition is activationless, while the activation of the $D \rightarrow A$ transition slows down with decreasing T) and would thus increase with decreasing temperature, against the experimental evidence.

$$P(T) \cong \frac{k_{01}k_{DA}}{(k_{10}+k_{DA})k_{AM}} = \frac{k_{01}\eta_{DA}\left\langle V_{DA}^2(T)\right\rangle}{[k_{10}+\eta_{DA}\left\langle V_{DA}^2(T)\right\rangle]\eta_{AM}\left\langle V_{AM}^2(T)\right\rangle} \approx \frac{k_{01}\eta_{DA}\left\langle V_{DA}^2(T)\right\rangle}{k_{10}\eta_{AM}\left\langle V_{AM}^2(T)\right\rangle} \qquad \textbf{(S3)}$$

The k_{DA} and k_{AM} rates involve the protein. The proportionality of these rate constants to the mean square electronic coupling, as well as the coupling dependence on temperature, are made explicit in **Equation S3**. P depends on $\left\langle V_{DA}^2(T)\right\rangle / \left\langle V_{AM}^2(T)\right\rangle$, and becomes proportional to such ratio if k_{10}>>k_{DA}. We make this further approximation to simplify the treatment. We also note that η_{AM} may depend on T, but this dependence is not considered in this study.

The probability P can be defined for each of the two spin states of the transferring electron. Since the $D \rightarrow A$ transition starts from Cu^{II}, $\left\langle V_{DA}^2(T)\right\rangle$ can generally depend on the electron spin. The $A \rightarrow M$ transition starts from a formally closed-shell Cu^{I}, but $\left\langle V_{AM}^2(T)\right\rangle$ may also depend on the spin of the transferring electron, due to partial charge-transfer character of the copper bond to the Met sulfur ligand.

Since the proteins were linked to the silver film through the Cys3 and Cys26 residues, in order to obtain relative $\left\langle V_{AM}^2(T)\right\rangle$ values at 290 K and 250 K, we analyzed the electron tunneling pathways from copper to both residues, using the same MD snapshots employed for the D → A transition as a simplifying approximation. We found that, on average, the electron tunneling to Cys3 is more than one order of magnitude faster than the one to Cys 26, based on the mean square electronic couplings (**Table S5**). The electron tunneling pathway analysis reported in **Table S6** shows that the Met121 ligand to copper is mainly involved in the electron transfer. Furthermore, about 20% and 3% of the strongest electronic

tunneling pathways involve His46 at 290 K and 250 K, respectively. We also found that the coupling between Cu and Cys3 is much weaker than that between Cu and Ru (compare **Tables S4** and **S5**).

Table S5. Mean-square electronic coupling decay factor (decay product) $\langle T^2 \rangle$, in (dimensionless) units of 10^{-13}, for the electron tunneling from copper to the cysteine residues (C3 and C26) linked to the silver film ($A \rightarrow M$ transition), at the indicated temperatures. We also report the mean-square decay product for the tunneling pathways to Cys3 through the M121 and H46 ligands to the copper.

	$\langle T_{Cu\text{-}C3}^2 \rangle$	$\langle T_{Cu\text{-}C3}^2 \rangle_{M121}$	$\langle T_{Cu\text{-}C3}^2 \rangle_{H46}$	$\langle T_{Cu\text{-}C26}^2 \rangle$
290 K	4.40	4.87	2.61	0.12
250 K	6.47	6.57	3.21	0.16

Table S6. Frequency of the dominant tunneling pathways between Cu and Cys3 (namely, the $A \rightarrow M$ transition, apart from the final step to the silver film) along the dynamic evolution of the His83 protein at the temperatures of 290 K and 250 K. The pathways involving the same residue sequence at the two temperatures are marked with the same color. Amino acid one-letter codes are used.

	**290 K**	**%**	**250 K**	**%**
1	Cu-M121-G9-Q8-I7-D6-V5-S4-C3	69.1	Cu-M121-G9-Q8-I7-D6-V5-S4-C3	91.6
2	Cu-H46-G9-Q8-I7-D6-V5-S4-C3	19.6	Cu-M121-F15-I7-D6-V5-S4-C3	5.3
3	Cu-M121-F15-I7-D6-V5-S4-C3	10.3	Cu-H46-G9-Q8-I7-D6-V5-S4-C3	3.0
4	Cu-H46-L33-N32-V5-S4-C3	0.5	Cu-H46-L33-N32-V5-S4-C3	0.1
5	Cu-H46-L33-N32-S4-C3	0.5		

Since the mean-square electronic coupling decay factors are proportional to the respective absolute values of the electronic couplings (to the extent to which the best pathway analysis approximately holds), inserting the data from the first columns of **Tables S4** and **S5** into **Equation S3**, we obtain the ratio $P(290\,\mathrm{K})/P(250\,\mathrm{K}) \cong 1.8$, in good agreement with the experimental results in **Fig. 1C** (since P is proportional to the voltage according to the kinetic model). It is worth noting that, although the approximations implicit

in the tunneling pathway method do not allow coupling calculations with less than order of magnitude accuracy, the broad statistical ensembles employed, the use of relative values, and the comparison with the experiments give support to the validity of this theoretical result and the underlying protein structure features analyzed above.

2.4 Model Calculations for Spin Dependent Transmission.

The model calculations, describing the temperature dependent spin polarization, are based on a description of two chiral molecules which are bridged by a single spin moment and mounted between two metallic leads, one which is ferromagnetic whereas the second is non-magnetic. The chiral molecules are represented by discrete sets of equivalent sites carrying an electron level. Each site is coupled to its nearest neighbor sites via a simple spin-conservative hopping and to its next nearest neighbor sites through a spin non-conservative hopping which ties together the spin-orbit interaction with the molecular chirality.

For the sake of proposing a model for charge transport through the complex of chiral molecules and a local spin moment, we begin by introducing the Hamiltonian:

$$\mathcal{H} = \mathcal{H}_L + \mathcal{H}_R + \mathcal{H}_{ML} + \mathcal{H}_{MR} - (\psi_{L\mathbb{M}}^{\dagger} \boldsymbol{V} \psi_{R1} + H.c.) + \mathcal{H}_{SQ}$$

$$\mathcal{H}_{SQ} = -g\mu_B(\boldsymbol{B} + \boldsymbol{B}_J) \cdot \boldsymbol{S} + \sum_n (\omega_n b_n^{\dagger} b_n - \boldsymbol{S} \cdot \mathcal{A}_n Q_n) + \sum_{ijk} \Phi Q_i Q_j Q_k.$$

Here, the term $\mathcal{H}_\chi = \sum_{\boldsymbol{k} \in \chi} \epsilon_{\boldsymbol{k}} \psi_{\boldsymbol{k}}^{\dagger} \psi_{\boldsymbol{k}}$, $\chi = L, R$, defines the electronic properties of the left (L) and right (R) leads, where the spinor $\psi_{\boldsymbol{k}}^{\dagger}(\psi_{\boldsymbol{k}})$ creates (annihilates) an electron in the lead χ at the energy $\epsilon_{\boldsymbol{k}}$, whereas $\mathcal{H}_{ML}$ and $\mathcal{H}_{MR}$ comprise the electronic structures of the left and right molecules, respectively. Here, the left molecule is described by the Hamiltonian:[18]

$$\mathcal{H}_{ML} = \sum_{m=1}^{\mathbb{M}} \psi_m^{\dagger} (\epsilon_m + \frac{1}{2} g\mu_B \boldsymbol{B} \cdot \boldsymbol{\sigma}) \psi_m - t \sum_{m=1}^{\mathbb{M}-1} (\psi_m^{\dagger} \psi_{m+1} + H.c.) + \lambda \sum_{m=1}^{\mathbb{M}-2} (i \psi_m^{\dagger} \boldsymbol{v}_m^{(+)} \cdot \boldsymbol{\sigma} \psi_{m+2} + H.c.)$$

and analogously for the right molecule. The spinor $\psi_m^{\dagger}(\psi_m)$ creates (annihilates) an electron at the site m with energy ϵ_m and Zeeman split $g\mu_B \boldsymbol{B} \cdot \boldsymbol{\sigma}/2$, where $\boldsymbol{\sigma}$ is the vector of Pauli matrices, generated by the external magnetic field $\boldsymbol{B}$. The second term describes hopping between nearest neighboring sites with rate t while the last term accounts for the spin-orbit

interaction with strength λ and chirality $\boldsymbol{v}_m^{\pm}$ in terms of hybridization between next-nearest neighbors. For more details, we refer to Ref 18.

The local spin moment is mounted between the two chiral molecules and we model its presence by a tunneling between the sites $\mathbb{M}$ and 1 in the left and right molecule, respectively, see fifth term in the expression for $\mathcal{H}$. The associated tunneling rate, $\boldsymbol{V}$, comprises spin-independent, V_0, and spin-dependent, V_1, contributions, such that $\boldsymbol{V} = V_0\sigma^0 + V_1\boldsymbol{S}\cdot\boldsymbol{\sigma}$.

The local spin moment is, finally, modelled by the Hamiltonian $\mathcal{H}_{SQ}$, where the first term accounts for the interaction with the external magnetic field $\boldsymbol{B}$ and the magnetic field induced by the electron current $\boldsymbol{B}_J$. The second term describes the ionic vibrations $\omega_n b_n^\dagger b_n$ in terms of the vibrational normal modes ω_n, and the electronically mediated interactions, $\mathcal{A}_n$, between the local spin moment and the ionic vibrations, where $Q_n = b_n + b_n^\dagger$ is the ionic quantum displacement operator. The last two terms, finally, provide anharmonic properties to the ionic motion, with strength Φ.

The current $J(t)$ across the interface between the two chiral molecules can be calculated as:[19]

$$J(t) = -2eIm(-i)\int_{-\infty}^{t}\{\langle\boldsymbol{V}_{\sigma\sigma'}(t)\boldsymbol{V}_{ss'}^\dagger(t')\rangle G_{R\sigma's}^{>}(t,t')G_{Ls'\sigma}^{<}(t',t) \\ - \langle\boldsymbol{V}_{ss'}^\dagger(t')\boldsymbol{V}_{\sigma\sigma'}(t)\rangle G_{R\sigma's}^{<}(t,t')G_{Ls'\sigma}^{>}(t',t)\}dt'$$

The expectation value, e.g., $\langle\boldsymbol{V}_{\sigma\sigma'}(t)\boldsymbol{V}_{ss'}^\dagger(t')\rangle$ can be decomposed according to

$$\langle\boldsymbol{V}_{\sigma\sigma'}(t)\boldsymbol{V}_{ss'}^\dagger(t')\rangle V_0^2\delta_{\sigma\sigma'}\delta_{ss'} + V_1^2\boldsymbol{\sigma}_{\sigma\sigma'}\cdot\langle\boldsymbol{S}_1(t)\boldsymbol{S}_1(t')\rangle\cdot\boldsymbol{\sigma}_{ss'} + V_0V_1(\langle\boldsymbol{S}_1(t)\rangle\cdot\boldsymbol{\sigma}_{\sigma\sigma'}\delta_{ss'} \\ + \langle\boldsymbol{S}_1(t')\rangle\cdot\boldsymbol{\sigma}_{ss'}\delta_{\sigma\sigma'})$$

which suggests that the current can be written $J = \sum_{i=0,1,2} J_i$, where

$$J_0(t) = 2eV_0^2 Resp\int_{-\infty}^{t}(\boldsymbol{G}_R^{>}(t,t')\boldsymbol{G}_L^{<}(t',t) - \boldsymbol{G}_R^{<}(t,t')\boldsymbol{G}_L^{>}(t',t))dt',$$

$$J_1(t) = 2eV_0V_1 Resp\int_{-\infty}^{t}\boldsymbol{\sigma}\cdot(\langle\boldsymbol{S}(t)\rangle(\boldsymbol{G}_R^{>}(t,t')\boldsymbol{G}_L^{<}(t',t) - \boldsymbol{G}_R^{<}(t,t')\boldsymbol{G}_L^{>}(t',t)) \\ +\langle\boldsymbol{S}(t')\rangle(\boldsymbol{G}_L^{<}(t',t)\boldsymbol{G}_R^{>}(t,t') - \boldsymbol{G}_L^{>}(t',t)\boldsymbol{G}_R^{<}(t,t')))dt',$$

$$J_2(t) = 2eV_1^2 Resp\int_{-\infty}^{t}(\boldsymbol{G}_L^{<}(t',t)\boldsymbol{\sigma}\cdot\langle\boldsymbol{S}(t)\boldsymbol{S}(t')\rangle\cdot\boldsymbol{G}_R^{>}(t,t')\boldsymbol{\sigma} - \boldsymbol{G}_R^{<}(t,t')\boldsymbol{\sigma}\cdot\langle\boldsymbol{S}(t')\boldsymbol{S}(t)\rangle \\ \cdot\boldsymbol{G}_L^{>}(t',t)\boldsymbol{\sigma})dt'.$$

The currents are, in these expressions, given in terms of the lesser and greater Green functions $\boldsymbol{G}_L^{</>}$ and $\boldsymbol{G}_R^{</>}$ of the sites $\mathbb{M}$ in the left molecule and 1 in the right, respectively.

Under the present construction, the chiral structures themselves do not generate spin selectivity. It is only through their interactions with the local spin moment such an effect takes place, if any. Hence, we can safely assume that the current J_0 does not provide any contribution to the spin selectivity since the local spin moment is absent from this expression. It should be noticed in this context that the Green functions for the left and right chiral structures are given in the atomic limit, that is, in absence of the local spin moments. This is justified since we are interested in whether a mechanism for spin selectivity can be achieved from the properties of the local moment and its coupling to the chiral environment. Moreover, it is justified to neglect possible spin-selectivity mechanisms, e.g., electron correlations[18] or spin-dependent electron-phonon interactions,[20] in order to resolve the hypothesis that transport through the chiral molecules is the source of a local magnetic field which acts on the spin moment and, hence, generates spin-selectivity.

To the lowest order in the interaction with the spin moment, then, the current is provided by J_1. It can be seen that this current depends explicitly on the magnitude of the spin moment, hence, it may be finite only whenever the expectation value of the spin moment is. We refine the analysis of this current by introducing the approximation $\boldsymbol{G}_\chi^{</>}(\omega) = (\pm i) f_\chi(\pm\omega)[-2Im\boldsymbol{G}_\chi^r(\omega)]$, where $f_\chi(\omega) = f(\omega - \mu_\chi)$ is the Fermi function at the chemical potential μ_χ. The Fourier transformed Green function $\boldsymbol{G}(\omega) = \int \boldsymbol{G}(t,t') e^{i\omega(t-t')} dt'$ is relevant under the assumption of stationary conditions. Furthermore, we are interested in the conditions that lead to a fixed local moment, wherefore, it is justified to assume that $\langle \boldsymbol{S}(t) \rangle = \langle \boldsymbol{S} \rangle$, such that the current can be written:

$$J_1(t) = \frac{4e}{\pi} V_0 V_1 \langle \boldsymbol{S} \rangle \cdot \int sp\boldsymbol{\sigma} Im\boldsymbol{G}_R^r(\omega) Im\boldsymbol{G}_L^r(\omega)(f_L(\omega) - f_R(\omega)) d\omega.$$

Although we focus on the lowest order contribution to the current that depends on the local moment, we notice that also the current J_2 depends on the local spin structure though the spin-spin correlation function $\langle \boldsymbol{S}(t) \boldsymbol{S}(t') \rangle$. However, this current adds an essentially negligible contribution to the spin-selectivity and is, therefore, omitted in the present discussion.

Next, we consider the properties of the local spin moment, which interacts with the electronic current through the induced magnetic field $\boldsymbol{B}_J$,[21] and with the ionic structure through the electronically mediated exchange $\mathcal{A}$.[22] These two electronically mediated fields can be written as:

$$\boldsymbol{B}_J(V) = \frac{V_0 V_1}{g\mu_B}\mathcal{K}(V), \tag{S4a}$$

$$\mathcal{A}(V) = -\frac{\lambda_{e\text{-}ph} V_1}{g\mu_B}\mathcal{K}(V), \tag{S4b}$$

where

$$\mathcal{K}(V) = -\frac{1}{2}\int (f_L(\omega) - f_R(\omega))sp\boldsymbol{\sigma}(Im\boldsymbol{G}_R^r(\omega)Re\boldsymbol{G}_L^r(\omega) + Re\boldsymbol{G}_L^r(\omega)Im\boldsymbol{G}_R^r(\omega))\frac{d\omega}{2\pi},$$

accounts for the spin-polarized current that passes through the system, and where $\lambda_{e\text{-}ph}$ is the parameter for the effective interaction between the electrons and the ionic motion. In terms of these fields, an effective mean-field model for the local spin moment can be written as

$$\mathcal{H}_S = -g\mu_B \boldsymbol{S} \cdot (\boldsymbol{B} + \boldsymbol{B}_J - \mathcal{A}\sum_n \langle Q_n \rangle), \tag{S5}$$

where $\langle Q_n \rangle$ denotes the expectation value of the displacement operator. Hence, while the induced field $\boldsymbol{B}_J$ depends only on the current flowing through the molecular complex, the induced field $B_{vib} = \mathcal{A}\sum_n \langle Q_n \rangle$ depends on both this current as well as the ionic vibrations that couple to the spin. In this sense, the former field has no temperature dependence whereas that latter has, something which will become crucial in our subsequent discussion.

The ionic motion is subject to anharmonic forces, which, here, are modelled in terms of the cubic interaction term $\Phi Q_i Q_j Q_k$. In linear response theory, the expectation value of the displacement operator, can be expanded in terms of the pertinent interaction Hamiltonian $\mathcal{H}_{int}(t)$ according to $\langle Q_n(t)\rangle \approx \langle Q_n(t)\rangle_0 + (-i)\int_{-\infty}^{t}\langle [Q_n(t), \mathcal{H}_{int}(t')]\rangle_0 dt'$, where $\langle Q_n(t)\rangle_0 = 0$ since it represents the displacement in the harmonic approximation. The second term, here, provides the contribution

$$(-i)\int_{-\infty}^{t}\langle [Q_n(t), \Phi Q_i Q_j Q_k(t')]\rangle_0 dt' = i\Phi \int D_n^r(t,t')\sum_i D_i^>(t',t')dt',$$

where the bare retarded and greater phonon Green functions are given by

$$D_n^>(t,t') = (-i)((1 + n_B(\omega_n))e^{-i\omega_n(t-t')} + n_B(\omega_n)e^{i\omega_n(t-t')}),$$

$$D_n^r(t,t') = 2\theta(t-t')sin\omega_n(t-t').$$

In terms of these expression, the linear response quantum displacement becomes time-independent and given by

$$\langle Q_n \rangle = -\frac{2\Phi}{\omega_n}\sum_i (1+2n_B(\omega_i)).$$

While the above calculation demonstrates that the ionic displacement may increase with temperature which, hence, leads to an increased vibrationally induced magnetic field acting on the local moment, it is not sufficient to generate a large difference in the induced field with the temperature increase. Therefore, we reconsider the phonon Green function in the anharmonic model, to lowest non-trivial order giving:

$$\begin{aligned} D_n^{(2)}(t,t') &= \frac{\Phi^2}{i^3}\sum\int \langle T(Q_iQ_jQ_k)(\tau)(Q_lQ_mQ_p)(\tau')Q_n(t)Q_n(t')d\tau d\tau' \\ &= i\Phi^2\sum_{ij}\int D_n(t,\tau)D_i(\tau,\tau')(D_j(\tau,\tau')D_n(\tau',t') + D_j(\tau',\tau')D_n(\tau,t'))d\tau d\tau' \\ &= \int D_n(t,\tau)(\Sigma_H(\tau)D_n(\tau,t') + \int \Sigma_F(\tau,\tau')D_n(\tau',t')d\tau')d\tau, \end{aligned}$$

where the self-energies

$$\Sigma_H(t,t') = i\Phi^2\sum_{ij}\int D_i(t,t')D_j(t',t')dt',$$
$$\Sigma_F(t,t') = i\Phi^2\sum_{ij} D_i(t,t')D_j(t,t'),$$

representing the Hartree and Fock contributions, respectively. Under the present conditions, only the former, the Hartree, contribution is considered. This can be justified since the temperature dependences of the two contributions are essentially the same.

Using standard methods, the Hartree self-energy becomes:

$$\Sigma_H = -2\Phi^2\sum_{ij}\frac{1}{\omega_i}coth(\beta\omega_j/2).$$

Hence, the retarded and greater second order corrections due to the Hartree contribution becomes:

$$\int \quad D_n^r(t,\tau)\Sigma_H^r D_n^r(\tau,t')d\tau = \Sigma_H\int (D_n^r(\omega))^2 e^{-i\omega(t-t')}\frac{d\omega}{2\pi},$$
$$\int \Sigma_H^r(D_n^r(t,\tau)D_n^>(\tau,t) + D_n^>(t,\tau)D_n^a(\tau,t))d\tau = \frac{i}{\omega_n}\Sigma_H coth\frac{\beta\omega_n}{2}.$$

Inserting the expansion of the phonon Green function, we obtain the displacement:

$$\langle Q_n \rangle = \quad -\frac{2\Phi}{\omega_n}\sum_{\nu}[1-\frac{1}{\omega_\nu}\Sigma_H+\frac{2}{\omega_n}(1+\frac{1}{\omega_\nu}\Sigma_H)\Sigma_H]coth\frac{\beta\omega_\nu}{2}.$$

References

[1] Phillips, J. C.; Braun, R.; Wang, W.; Gumbart, J.; Tajkhorshid, E.; Villa, E.; Chipot, C.; Skeel, R. D.; Kalé, L.; Schulten, K. Scalable Molecular Dynamics with Namd. *J. Comput. Chem.* **2005,** *26*, 1781-1802.

[2] Ryckaert, J. P.; Ciccotti, G.; Berendsen, H. J. C. Numerical Integration of the Cartesian Equations of Motion of a System with Constraints: Molecular Dynamics of N-Alkanes. *J. Comput. Phys.* **1977,** *23*, 327-341.

[3] Darden, T.; York, D.; Pedersen, L. Particle Mesh Ewald: An N·Log(N) Method for Ewald Sums in Large Systems. *J. Chem. Phys.* **1993,** *98*, 10089-10092.

[4] Kitayner, M.; Rozenberg, H.; Kessler, N.; Rabinovich, D.; Shaulov, L.; Haran, T. E.; Shakked, Z. Structural Basis of DNA Recognition by P53 Tetramers. *Mol. Cell* **2006,** *22*, 741-753.

[5] Balabin, I. A.; Hu, X. Q.; Beratan, D. N. Exploring Biological Electron Transfer Pathway Dynamics with the Pathways Plugin for Vmd. *J. Comput. Chem.* **2012,** *33*, 906-910.

[6] Humphrey, W.; Dalke, A.; Schulten, K. Vmd: Visual Molecular Dynamics. *J. Mol. Graph.* **1996,** *14*, 33-38.

[7] Beratan, D. N.; Onuchic, J. N.; Hopfield, J. J. Electron-Tunneling through Covalent and Noncovalent Pathways in Proteins. *J. Chem. Phys.* **1987,** *86*, 4488-4498.

[8] Onuchic, J. N.; Beratan, D. N. A Predictive Theoretical Model for Electron Tunneling Pathways in Proteins. *J. Chem. Phys.* **1990,** *92*, 722-733.

[9] Beratan, D. N.; Onuchic, J. N.; Betts, J. N.; Bowler, B. E.; Gray, H. B. Electron-Tunneling Pathways in Ruthenated Proteins. *J. Am. Chem. Soc.* **1990,** *112*, 7915.

[10] Beratan, D. N.; Betts, J. N.; Onuchic, J. N. Protein Electron Transfer Rates Set by the Bridging Seconday and Tertiary Structure. *Science* **1991,** *252*, 1285-1288.

[11] Beratan, D. N.; Betts, J. N.; Onuchic, J. N. Tunneling Pathway and Redox-State-Dependent Electronic Couplings at Nearly Fixed Distance in Electron-Transfer Proteins. *J. Phys. Chem.* **1992,** *96*, 2852-2855.

[12] Jones, M. L.; Kurnikov, I. V.; Beratan, D. N. The Nature of Tunneling Pathway and Average Packing Density Models for Protein-Mediated Electron Transfer. *J. Phys. Chem. A* **2002,** *106*, 2002–2006.

[13] Mizoguchi, T. J.; Di Bilio, A. J.; Gray, H. B.; Richards, J. H. Blue to Type 2 Binding. Copper(Ii) and Cobalt(Ii) Derivatives of a Cys112asp Mutant of Pseudomonas Aeruginosa Azurin. *J. Am. Chem. Soc.* **1992,** *114*, 10076-10078.

[14] Faham, S.; Mizoguchi, T. J.; Adman, E. T.; Gray, H. B.; Richards, J. H.; Rees, D. C. Role of the Active-Site Cysteine of Pseudomonas Aeruginosa Azurin. Crystal Structure Analysis of the Cuii(Cys112asp) Protein. *J. Biol. Inorg. Chem*. **1997,** *2*, 464-469.

[15] Crane, B. R.; Di Bilio, A. J.; Winkler, J. R.; Gray, H. B. Electron Tunneling in Single Crystals of Pseudomonas Aeruginosa Azurins. *J. Am. Chem. Soc.* **2001,** *123*, 11623-11631.

[16] Migliore, A.; Nitzan, A. Nonlinear Charge Transport in Redox Molecular Junctions: A Marcus Perspective. *ACS nano* **2011,** *5*, 6669-6685. Migliore, A.; Schiff, P.; Nitzan, A. On the Relationship between Molecular State and Single Electron Pictures in Simple Electrochemical Junctions. *Phys. Chem. Chem. Phys.* **2012,** *14*, 13746-13753.

[17] Migliore, A.; Schiff, P.; Nitzan, A. On the Relationship between Molecular State and Single Electron Pictures in Simple Electrochemical Junctions. *Phys. Chem. Chem. Phys.* **2012,** *14*, 13746-13753.

[18] Fransson, J. Chirality-Induced Spin Selectivity: The Role of Electron Correlations. *J. Phys. Chem. Lett.* **2019,** *10*, 7126-7132.

[19] Fransson, J.; Eriksson, O.; Balatsky, A. V. Theory of Spin-Polarized Scanning Tunneling Microscopy Applied to Local Spins. *Phys. Rev. B* **2010,** *81*, 115454.

[20] Fransson, J. Vibrational Origin of Exchange Splitting and Chiral-Induced Spin Selectivity. *Phys. Rev. B* **2020,** *102*, 235416.

[21] Fransson, J.; Ren, J.; Zhu, J. X. Electrical and Thermal Control of Magnetic Exchange Interactions. *Phys. Rev. Lett.* **2014,** *113*, 257201.

[22] Fransson, J.; Thonig, D.; Bessarab, P. F.; Bhattacharjee, S.; Hellsvik, J.; Nordstrom, L. Microscopic Theory for Coupled Atomistic Magnetization and Lattice Dynamics. *Phys. Rev. Mater*. **2017,** *1*, 074404.
